# Development and validation of real-time PCR for in-wood detection of *Ceratocystis ficicola*, the agent of canker, wood discoloration, and wilt in the common fig tree (*Ficus carica* L.)

**DOI:** 10.1128/aem.00428-26

**Published:** 2026-06-26

**Authors:** Valentina Lumia, Lorenzo Sciarroni, Giorgio Gusella, Daniele Del Corpo, Giuliano Manetti, Erica Cesari, Angela Brunetti, Giancarlo Polizzi, Massimo Pilotti

**Affiliations:** 1 Research Centre for Plant Protection and Certification (CREA-DC), Rome - Council for Agricultural Research and Economics of Italy (CREA) 468547 , Rome, Italy; 2 Department of Agriculture, Food and Environment, University of Catania 548299, Catania, Italy; Royal Botanic Gardens Kew, Surrey, United Kingdom

**Keywords:** fig tree, *Ceratocystis ficicola*, quarantine pathogen, diagnosis, real-time PCR, EvaGreen, SYBR Green

## Abstract

*Ceratocystis ficicola* (CF) is a fungus causing a lethal disease of fig trees characterized by bark canker, wood discoloration, and wilt. First reported in Japan in the 1970s, the pathogen was recently detected in Greece and Italy, raising concern among Mediterranean countries where fig is an important crop. Consequently, the European Plant Protection Organization (EPPO) placed the pathogen on the A2 list of quarantine organisms. Monitoring efforts have been hindered by difficulties in pathogen isolation and the lack of reliable diagnostic tools. To address these limitations, we developed and validated a real-time PCR assay using the intercalating dyes EvaGreen and SYBR Green with primers targeting the ITS1 region of the ribosomal RNA operon. Amplification efficiency and selectivity were assessed using serial dilutions of CF genomic DNA spiked with various wood extracts. Efficiencies ranged from 95.7% to 102.6%, demonstrating no inhibition by host tissues. The EvaGreen assay consistently detected 5 fg of genomic DNA per reaction, with an operational limit of detection (LoD) of 10 fg (Ct 35–36), while the SYBR Green assay achieved an operational LoD of 3 fg. Cloning the CF ITS region into a plasmid vector indicated that these LoDs correspond to ~123 and ~37 target copies. The assay showed complete specificity against *C. platani* and 49 additional non-target fungi and full inclusivity for 8 CF isolates. Validation on 114 samples from artificially inoculated, naturally infected, and non-infected trees confirmed optimal diagnostic performance. This assay provides a sensitive and robust tool for detecting and monitoring CF in fig.

**IMPORTANCE:**

Diagnostic assessments for pathogenic microbes are fundamental to medical and plant pathology, supporting curative and preventive diagnosis, and enabling detailed investigations of disease biology, epidemiology, and resistance. *Ficus carica* (fig) is a key fruit crop in the Mediterranean basin and a characteristic component of its rural landscapes, where it has been cultivated since the early Neolithic. *Ceratocystis ficicola* (CF) is a fungus causing lethal bark canker and wilt of fig in Japan since the 1970s and was recently detected in Greece and Italy, raising concern among Mediterranean National Plant Protection Organizations, including those of major non-EU producers. These developments prompted the European Plant Protection Organization (EPPO) to place the pathogen on the A2 list of quarantine organisms, thereby laying the groundwork for mandatory phytosanitary measures. However, effective monitoring has been hampered by difficulties in pathogen isolation and the absence of reliable diagnostic tools. The severe damage caused by CF, its rapid local and long-distance spread, and its poor eradicability highlight the urgent need for a robust integrated diagnostic framework in which real-time PCR plays a central role in disease surveillance. This study represents an initial step toward that goal.

## INTRODUCTION

The fig tree (*Ficus carica* L.), a member of the Moraceae, is among the earliest domesticated fruit crops and is indigenous to the Middle East and Asia Minor (1, 2). *Ficus carica* is a notably resilient species, exhibiting high tolerance to drought and heat, and the capacity to grow across a wide range of soil conditions (2, 3). Owing to its ecological plasticity, fig cultivation is viable even in low-input or marginal agricultural areas, contributing to the recovery and use of otherwise unproductive lands.

Historically rooted in the Mediterranean Basin, fig cultivation has expanded across Asia and the Americas; however, global production remains largely concentrated in Mediterranean countries, with Turkey as the leading producer (https://worldpopulationreview.com/country-rankings/fig-production-by-country).

In Japan, a destructive disease of fig emerged in the 1970s, characterized by bark canker, wood necrosis, xylem dysfunction in roots and stems, and consequent canopy wilting that frequently culminated in tree death (4–9). The causal agent was initially identified as *Ceratocystis fimbriata* Ellis & Halsted (4) and later reclassified as *Ceratocystis ficicola* Kajitani & Masuya (10) (hereafter CF). This taxonomic revision established CF as a new species that was included in the Asian-Australian Clade (AAC) of the *Ceratocystis* complex (11, 12).

From the earliest outbreaks, it became clear that the severe impact of the disease was attributable not only to the pathogen’s high intrinsic virulence but also to several epidemiological characteristics that amplified its spread and persistence.

CF, similar to other members of the genus *Ceratocystis*, exploits fresh wounds for host invasion and persists predominantly as a soilborne pathogen, making its eradication particularly challenging. Dispersal pathways can be broadly categorized into short- and long-distance mechanisms. Short-distance spread is thought to occur through several natural processes, including the movement of fungal propagules via runoff water from infected to healthy trees and the windborne transport of contaminated insect frass. In Japan, the ambrosia beetle *Euwallacea interjectus* (Blandford) has been documented to heavily infest fig trees, carry CF on its elytra, and promote pathogen colonization by excavating galleries within the vascular tissue (6, 7, 13, 14).

Human-mediated pathways may also contribute substantially to local spread, although direct evidence remains limited. Contaminated ploughing machinery is suspected to facilitate soil-to-soil transfer of inoculum while inflicting root wounds conducive to infection. Likewise, pruning tools contaminated during canopy management may enable pathogen entry through fresh cuts (15).

Long-distance dissemination is presumed to be primarily anthropogenic and associated with the movement of infected or contaminated nursery stock. Potted plants—through infected tissues or infested growing media—represent the highest-risk pathway, followed by bare-rooted plants and unrooted cuttings. The trade in such materials has been implicated in the spread of CF across multiple fig-growing regions in Japan (7, 10) and is likely responsible for introductions into countries of the European Union (16).

Between 2018 and 2020, CF was isolated from diseased fig orchards in the Attica region and on the island of Euboea, marking its first detection within the European Union (15). Affected trees exhibited canopy thinning and foliar chlorosis, along with bark cankers and cracking, and wood discoloration at the basal portion of the stem. Pronounced root necrosis further indicated that infections likely originated in the soil before progressing upward into the aerial corm. Tree mortality was recorded as a frequent outcome in advanced stages of disease development (15).

Subsequently, in 2021 and 2022, CF was recovered from symptomatic fig trees in various areas of Southern Italy—specifically Sicily and Apulia—with the first molecular identification of CF isolates achieved in Sicily (17–20). Internal and external symptoms were similar to those described in Greece. Notably, in Sicily, the pathogen was also isolated from adults of the xylophagous beetle *Cryphalus dilutus*, a known fig-associated scolytine. The recovery of CF from beetles emerging from wood galleries of infected trees strongly suggests that *C. dilutus* may function as a local vector, facilitating pathogen movement within and potentially between distinct host plants (19–21).

Reports from Brazil further highlight the susceptibility of fig to *Ceratocystis* species outside the Mediterranean basin. In South America, infections on fig have been attributed not to CF but to *C. manginecans*, a member of the Latin American Clade (LAC), South American subclade of the *C. fimbriata* complex (22–24). The virulence of *C. manginecans* toward fig underscores the need for stringent phytosanitary measures to prevent its introduction into the EU, where it would represent an additional threat to fig cultivation.

The current diagnostic workflow for CF relies primarily on isolating fungal cultures from discolored woody tissues or soil, followed by microscopic examination of perithecia and conidia and Sanger sequencing of taxonomically informative loci (ITS, TEF1-α, and RPB2). Although sequencing provides reliable species-level identification, culture-based isolation remains challenging. As previously reported, CF colonies are frequently overgrown or suppressed by rapidly expanding co-inhabiting fungi commonly associated with diseased fig tissues (15, 20). The fig-twig baiting method improves recovery rates, yet perithecial development on the twigs requires 7–10 days, and definitive identification still depends on subsequent fungus culturing and sequencing. Given the broad morphological overlap among *Ceratocystis* species, DNA sequencing is essential for species delimitation, whereas microscopic traits remain useful primarily for genus-level resolution.

Some molecular assays have been proposed to circumvent the limitations of culture-based diagnostics. A CF-specific TaqMan Real-Time PCR assay (25) and a LAMP assay applicable to both plant tissues and soil (26) were among the earliest molecular tools developed. More recently, a genus-level TaqMan assay (27) was applied to CF-infected fig material. However, these molecular methods have not undergone extensive performance validation, and cross-reactivity with non-target fungi was also reported (27). Therefore, lack of validation, as well as the risk of false positives, would limit their applicability for reliable regulatory diagnostics.

In light of these constraints, we developed a new CF-specific real-time PCR assay and conducted a comprehensive validation of its performance characteristics, including amplification efficiency, selectivity, analytical sensitivity, analytical specificity, and diagnostic accuracy. Repeatability and reproducibility were rigorously assessed similar to the approach previously adopted for the real-time PCR assays targeting *Ceratocystis platani* (28, 29). This work provides a robust molecular framework for reliable detection of CF in wood, addressing a critical need for rapid and accurate diagnostics in areas at risk of pathogen establishment and spread.

## MATERIALS AND METHODS

### Fungal isolation

Wood and bark tissues were collected from symptomatic and asymptomatic fig trees in Sicily, Apulia, and Latium. Samples were sectioned to expose internal tissues, and small fragments (0.5–2 mm) were dissected from discolored/necrotic areas corresponding to fresh reaction zones, as well as from healthy wood and bark. Tissue fragments were briefly surface-sterilized by passing through a flame and plated on potato dextrose agar (PDA; Oxoid, Thermo Fisher Scientific, Waltham, MA, USA) supplemented with 300 mg L⁻¹ streptomycin. For each sample, 36 fragments were plated. Plates were incubated at 25°C in the dark. Emerging colonies were sub-cultured by transferring small mycelial fragments from colony margins onto fresh PDA plates and maintained at 5°C on PDA slants.

For long-term preservation of CF isolates, twig segments from a healthy fig tree (0.5–1 cm diameter, 7–8 cm length) were autoclaved twice, 72 h apart (first at 90°C, then at 121°C for 20 min each), and placed in 50 mL vials containing 9 mL PDA. Each vial was inoculated with fungal plugs, incubated at 25°C for 12 days, and stored at 7°C.

### Inoculation trials

Inoculation trials were conducted to obtain artificially infected fig and plane trees for use as sources of wood samples in the validation of diagnostic sensitivity and specificity. Two CF isolates were used: C1355 from Japan (kindly provided by T.C. Harrington, Iowa State University, USA) and CPC 44213 (= CBS 149669) from Sicily, Italy (kindly provided by G. Polizzi, University of Catania, Italy). Non-target fungi were also included in the trials: *Botryosphaeria dothidea* (CREA-DC TPR FIG.7), *Neofusicoccum parvum* (CREA-DC TPR FIG.6), and *N. vitifusiforme* (CREA-DC TPR FIG.5) isolated from declining fig trees in Apulia (Italy), and *C. platani*, isolated from a canker stain-affected *Platanus × acerifolia* tree in Rome, Italy (collection code: CREA-DC TPR CP23). Cross-inoculations were performed by inoculating *C. platani* on fig and CF, isolate CPC 44213, on plane trees.

Fig trees, supplied by Caforio Vivai (Palagiano [TA] Apulia, Italy), and plane tree seedlings, supplied by Umbraflor (Spello [PG], Umbria, Italy), were inoculated at the third year of age. Fig and plane trees were kept in 23 L and 6.5 L pots, respectively. The pot substrate was Radicom (Vigorplant [LO], Lombardy, Italy), which contained a mixture of peat moss, black peat, marsh peat, and vegetable compost. Humus was present in the form of humic and fulvic acids with a water pH of 6–6.5.

Inoculations were performed on the basal part of the stem at 15–20 cm above the soil surface. Rectangular PDA–mycelium plugs (16–18 × 3–4 mm for fig; 12–14 × 3 mm for plane trees) were excised from 15-day-old *Ceratocystis* colonies and 8-day-old Botryosphaeriaceae colonies. Bark flaps of corresponding size were generated by cutting the bark top-down through the cambium with a sterile razor blade and gently lifting the bark to expose the xylem. Mycelial plugs were applied directly to the xylem, and the bark flap was then gently set on the plug. Each inoculation site was covered with a sterile cotton pad wetted with 3.5 mL sterile water and wrapped with a strip of sterile aluminum foil that was taped to the stem along its upper and lower margins. Covers were removed after 30 days.

Five biological replicates were used for inoculation with each CF isolate, whereas four replicates were used for each Botryosphaeriaceae isolate and for *C. platani*. Four fig trees inoculated with sterile PDA served as negative controls, and five plane trees were inoculated with CF.

Symptom development was monitored visually until fig trees approached mortality or for a maximum of 3 months, after which trees were destructively sampled. Longitudinal and transverse sections of stems were examined to assess the extent of wood necrosis, which is typically associated with colonization by *Ceratocystis* spp. and Botryosphaeriaceae.

All inoculations were carried out in the quarantine laboratory, and plants were subsequently maintained under observation in the quarantine greenhouse at the Research Centre for Plant Protection and Certification (CREA-DC) in Rome.

For CF-inoculated trees, six to seven 90-mg wood aliquots were collected per stem. Three aliquots were taken from the uniform, darkly discolored necrotic streak (i.e., at the inoculation point and near the two ends of this part of the streak). Beyond this zone, where necrosis extended basipetally and acropetally as threadlike discoloration indicative of a more limited pathogen spread, additional aliquots were collected every 3 cm: one downward and two to three upward, depending on the length of the streak. In the uppermost part of the threadlike streak, where an aliquot was nevertheless collected, necrosis was only faintly visible as sparse, minute, dark points in transverse section. In total, 35 and 34 aliquots were collected from trees inoculated with the Japanese and Sicilian CF isolates, respectively, and processed by real-time PCR.

For non-target fungal inoculations and PDA controls, one pooled aliquot (90 mg) was obtained per tree. In fungi-inoculated trees, tissue was collected from two distinct points within discolored wood; in PDA controls, tissue was collected from healthy-appearing wood adjacent to the inoculation site

### DNA extraction, PCR amplification, Sanger and PacBio sequencing, and cloning

DNA was extracted from fig wood tissue—either healthy or discolored and fungus-infected—by powdering samples in an autoclaved mortar with liquid nitrogen. Depending on the starting material, either the DNeasy Plant Mini Kit (70–100 mg tissue) or the DNeasy Plant Maxi Kit (700 mg tissue) (Qiagen, Hilden, Germany) was used following the manufacturer’s instructions. The higher input (700 mg) was used to obtain sufficient DNA for standard curve experiments in which fungal genomic DNA (gDNA) was tested in serial dilutions spiked with a fixed amount of CF-uninfected wood extract. The DNeasy PowerClean Pro Cleanup Kit (Qiagen) was also used after DNA extraction to remove potential PCR inhibitors.

Genomic DNA of all non-target and target fungal isolates was obtained from 100 or 700 mg of mycelium, depending on downstream requirements. Mycelium was harvested aseptically using sterile pipette tips from axenic cultures actively growing on sterile cellophane discs (BIO-RAD, Hercules, CA, USA) placed on PDA. Samples were ground in liquid nitrogen and processed using either the DNeasy Plant Mini (100 mg) or Maxi (700 mg) Kit (Qiagen) according to the manufacturer’s instructions.

The complete ITS region of the ribosomal RNA operon, fragments of beta-tubulin 2 (TUB2), and translation elongation factor 1-alpha (TEF1-α) of target and non-target fungal isolates were amplified in endpoint PCR on a T100 Thermal Cycler (Bio-Rad). Reactions (50 μL) contained 0.2 μM (ITS and TUB2) and 0.3 μM (TEF1-α) of each primer, 2.5 U High Fidelity Platinum Taq DNA Polymerase (Invitrogen, Thermo Fisher Scientific, Waltham, MA, USA), 0.2 mM (ITS), and 0.3 mM (TUB2 and TEF1-α) of each dNTP, and the supplied reaction buffer. All details on primers and thermal cycling for the amplifications are reported in Table S1 and references therein (30–36).

PCR products were visualized by agarose gel electrophoresis, excised, and purified using the ISOLATE II PCR and Gel Kit (Meridian Bioscience, Cincinnati, OH, USA). All fungal amplicons were sequenced bidirectionally via Sanger sequencing (Eurofins Genomics Europe Sequencing, Ebersberg, Germany). Newly generated sequences were compared with GenBank accessions using the blastn suite on the NCBI server (https://blast.ncbi.nlm.nih.gov/Blast.cgi?PROGRAM=blastn&PAGE_TYPE=BlastSearch&LINK_LOC=blasthome).

For some CF isolates (C1355, CREA-DC TPR CF Sic2, and CREA-DC TPR CF Sic5), it was not possible to obtain readable Sanger chromatograms from direct sequencing of the ITS region PCR products. Therefore, these PCR products were cloned into the pGEM-T Easy vector using the pGEM-T and pGEM-T Easy Vector Systems (Promega Italia s.r.l., Milan, Italy), following the manufacturer’s instructions.

To investigate a suspected allelic variation in the ITS region of CF, genomic DNA from isolates C1355 and CPC 44213 (= CBS 149669) was submitted to Biomarker Technologies (BMKGENE, Beijing, China) for library preparation and full-length ITS sequencing of ITS1F/ITS4 primers on the PacBio Sequel II platform in circular consensus sequencing (CCS) mode. Bioinformatic processing and downstream analyses were performed by BMKGENE.

### Design of primers for real-time PCR based on the ITS region of *Ceratocystis ficicola*

Primer design was performed as follows. All ITS region sequences of CF isolates available at the National Center for Biotechnology Information (NCBI, https://www.ncbi.nlm.nih.gov/) at the time of the analysis (AB576637, AB576865-AB576867, NR_119410, OQ335969, OQ329983, KY685076, KY306683, and OQ990098-OQ990099) were downloaded and aligned with MAFFT (EMBL-EBI server, https://www.ebi.ac.uk/jdispatcher/msa/mafft). Primers were designed manually in the conserved nucleotide stretches in the ITS1 region and were as follows: Fwd-ITS1, 5′-TTCCCACTACCAGCAGYATAATTCTTC-3′ (positions 91–117 on the reference sequence AB576867) and Rev-ITS1, 5′-CCACTCAGCAATGAAATCAAATTTCTAAC-3′ (positions 154–182 on the same reference sequence). The expected size of the amplicon was 92 bp.

NCBI Nucleotide blast (https://blast.ncbi.nlm.nih.gov/Blast.cgi?PROGRAM=blastn&PAGE_TYPE=BlastSearch&LINK_LOC=blasthome) was used to check the degree of similarity between the selected primer sequences and other biological sequences from related and unrelated organisms.

### First-phase optimization of real-time PCR

The CF isolate C1355 was used for all experiments aimed at optimizing, establishing, and validating the real-time PCR assay. Amplifications were performed on a CFX96 Real-Time PCR Detection System (Bio-Rad) using SsoFast EvaGreen Supermix (Bio-Rad), which contains EvaGreen, a saturating double-stranded DNA-binding fluorescent dye. Reactions were carried out in 30 μL volumes containing 15 μL of Supermix and 0.5 μM of each primer. The cycling protocol consisted of an initial denaturation at 96°C for 3 min; 40 two-step cycles of 95°C for 15 s and the selected annealing/extension temperature for 30 s; and a final extension at 72°C for 5 min.

To determine the optimal annealing/extension temperature, an initial gradient PCR spanning 55.7°C–64.5°C was performed using 0.5 pg of CF gDNA. Based on these results, narrower gradients (59°C–63°C and 62°C–65°C) were subsequently tested to refine temperature selection. No-template controls were included in each run. To check the specificity of the primers to the ITS region and exclude any potential to reciprocally anneal at 3′ and give primer-dimers (a possible false-positive signal in any dye-based real-time PCR), we included a dissociation run with a temperature increase by 0.1°C, which enabled a melt curve to be generated. Each temperature set was tested twice with three technical replicates.

To further exclude reciprocal primer annealing and dimer formation, additional EvaGreen reactions lacking template DNA were run at the chosen annealing/extension temperature. These assays included melt curve analysis and were conducted twice with six technical replicates.

The amplification product generated under the selected annealing/extension temperature was sequenced to confirm its identity as the intended target amplicon.

A technical approach based on SYBR Green chemistry, using the SsoAdvanced Universal SYBR Green Supermix (Bio-Rad), was also evaluated and applied to selected steps of the workflow (see below).

Unless otherwise specified, all procedures described below and all results presented were generated using the EvaGreen-based supermix; experiments performed with the SYBR Green–based supermix are explicitly indicated.

### Efficiency of the amplification process in relation to the wood matrix

To assess the extraction approach that most effectively reduced potential PCR inhibition caused by necrotic fig wood, we evaluated the amplification performance by supplementing two CF gDNA inputs (500 fg and 25 fg per reaction) with 1 and 3 μL of DNA extracts obtained separately from discolored wood of two fig trees naturally infected with fungi other than CF. Wood had been extracted with the DNeasy plant kit alone or in combination with the DNeasy PowerClean Pro Cleanup Kit. Overall, this design resulted in 8 treatment combinations per tree-wood-extract (thus a total of 16 treatments) besides the non-spiked controls. Each treatment was evaluated with five technical replicates

To assess amplification efficiency and linearity, and to obtain a preliminary estimate of the limit of detection (LoD), standard curves were generated using fivefold serial dilutions of CF gDNA ranging from 78.12 pg to 200 ag (nine dilution points). To evaluate the effect of fig wood matrices on amplification performance, additional standard curves were produced using the same dilution series, but each reaction was supplemented with 2 µL of fig wood DNA extract. Three types of wood extracts were tested independently: (i) healthy-looking CF-uninfected wood, (ii) freshly necrotized wood, and (iii) dried necrotized wood (the latter two infected with fungi other than CF). The necrotized wood extracts used for the standard curve experiments were obtained from samples collected in the Latium region (where CF is not present), and their fungal communities were assessed by culture-based isolation and sequencing (as described above).

Nine fivefold serial dilutions of a plasmid containing the complete CF ITS region (ranging from 3.1 pg to 8 ag of DNA) were amplified to construct standard curves. Amplification performance was evaluated using both circular and PstI-linearized plasmid templates (37).

In total, six experimental setups were evaluated; each setup was performed in three independent runs, and each dilution point was assayed in triplicate. No template reactions and reactions containing gDNA extracted from CF-uninfected wood of the fig tree were always included as controls. In each standard curve experiment, the dissociation run with a temperature increase by 0.1°C was included.

We evaluated potential variations in the melting temperature of the target amplicon as a function of (i) the CF isolate, (ii) the amount of fungal gDNA, and (iii) the presence of wood extract. To this end, we amplified 1 ng, 1 pg, and 10 fg of gDNA from both the Japanese and Sicilian CF isolates (CF1355 and CPC 44213), either unspiked or spiked with 2 µL necrotic wood extract. Each treatment included four technical replicates. The experiment was conducted twice. The dissociation run was as described above.

The same experiment described above, used to assess potential variations in the melting temperature of the target amplicon as a function of CF isolate, fungal gDNA amount, and the presence of wood extract, was also performed using a SYBR Green-based supermix.

### Real-time PCR: the performance parameters

#### Analytical sensitivity

The LoD was defined as the lowest DNA quantity per PCR (DQP-ASe, DNA Quantity per PCR—Analytical Sensitivity) (28) that was detected consistently at a Ct of approximately 35–36, which was set as the upper acceptable threshold cycle range. To determine whether wood-derived matrices affected the LoD, four template types were tested by spiking, as described for the standard-curve experiments: (i) CF gDNA alone; (ii) CF gDNA supplemented with 2 µL of DNA from healthy-looking wood; (iii) with DNA from freshly necrotized wood; and (iv) with DNA from dried necrotized wood. Two LoD values—5 and 10 fg of CF gDNA per reaction—were assessed. In parallel, 500 and 200 ag were the LoD tested with a plasmid containing the CF ITS region.

SYBR Green-based supermix was used to test 3 and 5 fg of CF gDNA per reaction, supplemented with 2 µL of DNA from freshly necrotized wood.

Repeatability and reproducibility of the DQP-ASe were evaluated through three independent experiments for each template type at both DNA quantities. In each experiment, eight technical replicates per treatment were performed, resulting in a total of 24 technical replicates for each template type × DNA quantity × chemistry combination.

Evaluation of the target copy number at the LoD was performed by calculating the copy number of the cloned amplicon target-containing ITS region at the limit of detection, namely at a Ct of approximately 35–36. Calculation was performed using the DNA Copy Number Calculator (https://www.technologynetworks.com/tn/tools/copynumbercalculator).

#### Analytical specificity: exclusivity and inclusivity

Analytical specificity was assessed in terms of exclusivity and inclusivity, defined as the ability of the assay to yield negative results for non-target organisms (exclusivity) and positive results for target organisms (inclusivity). For each parameter, the performance was expressed as the proportion of non-target isolates not amplified over the total number tested (exclusivity) and the proportion of target isolates detected over the total tested (inclusivity); percentages were calculated by multiplying each ratio by 100.

Exclusivity was evaluated using 50 non-target fungal taxa isolated from fig and olive woody tissues collected during surveys in the Latium, Sicily, and Apulia regions (Italy), and also including *Ceratocystis platani* (Table 1). Inclusivity was assessed using eight CF isolates obtained from diseased fig trees in Sicily (Table 2). Identification of the fungi was carried out based on sequences of taxonomically informative DNA loci generated in the present study, as described above. For a subset of fungal isolates, molecular identification relied on sequence data previously obtained and reported in earlier studies (17, 20, 38, 39) (Tables 1 and 2).

**TABLE 1:** Non-target fungi isolated from healthy-looking and discolored wood of fig and olive trees and used to validate the exclusivity of the real-time PCR method of this study^*e*^

Fungal isolate code^*f*^	Geographical origin of the isolates	Identification in nucleotide BLAST	Accession no.	Best matching subjects sharing 100% coverage and 99%–100% nucleotide identity with the fig queries identified in a single locus (accession no.)	Result of detection of 500 pg gDNA^*a*^
CREA-DC TPR FIG.3	Apulia, Italy	*Diaporthe* sp.	PX613668 (ITS)	*Diaporthe foeniculina* (OL477412.1)	N.D.
CREA-DC TPR FIG.4	Apulia, Italy	*Diplodia seriata*	PX613669 (ITS)	*Diplodia seriata* (MT587384.1)	N.D.
CREA-DC TPR FIG.5	Apulia, Italy	*Neofusicoccum vitifusiforme*	PX613670 (ITS), PX995852 (TEF1-α), PX995869 (TUB2)		N.D.
CREA-DC TPR FIG.6	Apulia, Italy	*Neofusicoccum parvum*	PX613671 (ITS), PX995853 (TEF1-α), PX995870 (TUB2)		N.D.
CREA-DC TPR FIG.7	Apulia, Italy	*Botryosphaeria dothidea*	PX613672 (ITS), PX995854 (TEF1-α), PX995871 (TUB2)		N.D.
CREA-DC TPR FIG.8	Apulia, Italy	*Fusarium* sp.	PX613673 (ITS)	*Fusarium* sp. (AF178404.1)	N.D.
CREA-DC TPR FIG.10	Latium, Italy	*Apiospora arundinis*	PX613674 (ITS), PX995855 (TEF1-α), PX995872 (TUB2)		N.D.
CREA-DC TPR FIG.11	Latium, Italy	*Alternaria* sp.	PX613675 (ITS)	*Alternaria alternata* (MH862799.1)	N.D.
CREA-DC TPR FIG.12	Latium, Italy	*Epicoccum* sp.	PX613676 (ITS)	*Epicoccum nigrum* (PQ678942.1)	N.D.
CREA-DC TPR FIG.14	Latium, Italy	*Pseudopithomyces* sp.	PX613677 (ITS)	*Pseudopithomyces* sp. (OQ793651.1)	N.D.
CREA-DC TPR FIG.16	Latium, Italy	*Diplodia mutila*	PX613678 (ITS), PX995856 (TEF1-α), PX995873 (TUB2)		N.D.
CREA-DC TPR FIG.17	Latium, Italy	*Biscogniauxia mediterranea*	PX613679 (ITS), PX995857 (TEF1-α), PX995874 (TUB2)		N.D.
CREA-DC TPR FIG.20	Latium, Italy	*Fusarium* sp.	PX613680 (ITS)	*Fusarium* sp. (OP834780.1)	N.D.
CREA-DC TPR FIG.21	Latium, Italy	*Dothiorella viticola*	PX613681 (ITS), PX995858 (TEF1-α), PX995875 (TUB2)		N.D.
CREA-DC TPR FIG.22	Latium, Italy	*Clonostachys* sp.	PX613682 (ITS)	*Clonostachys rosea* (PP938457.1)	N.D.
CREA-DC TPR FIG.23	Latium, Italy	*Neodidymelliopsis* sp.	PX613683 (ITS)	*Neodidymelliopsis* sp. (OP179088.1)	N.D.
CREA-DC TPR FIG.24	Latium, Italy	*Diaporthe* sp.	PX613684 (ITS)	*Phomopsis* sp. (JX515731.1)	N.D.
CREA-DC TPR FIG.27	Latium, Italy	*Alternaria* sp.	PX613685 (ITS)	*Alternaria infectoria* (MN313351.1)	N.D.
CREA-DC TPR FIG.31	Latium, Italy	*Sardiniella* sp.	PX613686 (ITS), PX995859 (TEF1-α), PX995876 (TUB2)		N.D.
CREA-DC TPR FIG.35	Latium, Italy	*Neofusicoccum parvum*	PX613687 (ITS), PX995860 (TEF1-α), PX995877 (TUB2)		N.D.
CREA-DC TPR FIG.36	Latium, Italy	*Diaporthe* sp.	PX613688 (ITS)	*Diaporthe eres* (KC343074.1)	N.D.
CREA-DC TPR FIG.37	Latium, Italy	*Apiospora* sp.	PX613689 (ITS), PX995861 (TEF1-α), PX995878 (TUB2)		N.D.
CREA-DC TPR FIG.38	Latium, Italy	*Diaporthe* sp.	PX613690 (ITS)	*Diaporthe amygdali* (KC343019.1)	N.D.
CREA-DC TPR FIG.40	Latium, Italy	*Peniophora* sp.	PX613691 (ITS)	*Peniophora lycii* (MH857357.1)	N.D.
CREA-DC TPR FIG.44	Latium, Italy	*Apiospora marii*	PX613692 (ITS), PX995862 (TEF1-α), PX995879 (TUB2)		N.D.
CREA-DC TPR FIG.45	Latium, Italy	*Diaporthe cinerascens*	PX613693 (ITS), PX995863 (TEF1-α), PX995880 (TUB2)		N.D.
CREA-DC TPR FIG.46	Latium, Italy	*Geosmithia pallida*	PX613694 (ITS), PX995864 (TEF1-α), PX995881 (TUB2)		N.D.
CREA-DC TPR FIG.49	Latium, Italy	*Lasiodiplodia* sp.	PX613695 (ITS), PX995865 (TEF1-α), PX995882 (TUB2)		N.D.
CREA-DC TPR FIG.51	Latium, Italy	*Botryosphaeria dothidea*	PX613696 (ITS), PX995866 (TEF1-α), PX995883 (TUB2)		N.D.
CREA-DC TPR FIG.52	Sicily, Italy	*Fusarium* sp.	PX613697 (ITS)	*Fusarium* sp. (OR553244.1)	N.D.
CREA-DC TPR FIG.56	Sicily, Italy	*Fusarium* sp.	PX613698 (ITS)	*Fusarium* sp. (PP551988.1)	N.D.
CREA-DC TPR FIG.62	Sicily, Italy	*Fusarium* sp.	PX613699 (ITS)	*Fusarium solani* (KF030977.1)	N.D.
CREA-DC TPR FIG.63	Sicily, Italy	*Clonostachys* sp.	PX613700 (ITS)	*Clonostachys rosea* (PP976547.1)	N.D.
CREA-DC TPR FIG.64	Sicily, Italy	*Fusarium* sp.	PX613701 (ITS)	*Fusarium waltergamsii* (PV366669.1)	N.D.
CREA-DC TPR FIG.65	Sicily, Italy	*Fusarium* sp.	PX613702 (ITS)	*Fusarium solani* (PV124118.1)	N.D.
CREA-DC TPR FIG.66	Sicily, Italy	*Neofusicoccum parvum*	PX613703 (ITS), PX995867 (TEF1-α), PX995884 (TUB2)		N.D.
CREA-DC TPR FIG.70	Latium, Italy	*Dothiorella viticola*	PX613705 (ITS), PX995868 (TEF1-α), PX995885 (TUB2)		N.D.
CREA-DC TPR FIG.71	Latium, Italy	*Botryosphaeria dothidea*	PX613706 (ITS)	*Botryosphaeria* sp. (OP970623.1)	N.D.
FUS C8C = CPC 44200^*b*^	Sicily, Italy	*Neocosmospora perseae*	PP094711 (ITS), PP105770 (TEF1-α), PP125184 (RPB2)		N.D.
FUS.C11A = CPC 44202^*b*^	Sicily, Italy	*Neocosmospora bostrycoides*	PP094708 (ITS), PP105767 (TEF1-α), PP125181 (RPB2)		N.D.
CREA-DC TPR OL.193^*c*^	Latium, Italy	*Neoscytalidium* dimidiatum	PZ012350 (ITS), PZ016878 (TEF1-α), PZ016879 (TUB2)		N.D.
CREA-DC TPR OL.297^*c*^	Latium, Italy	*Apiospora rasikravindrae*	PX903889 (ITS), PX894992 (TEF1-α), PX894995 (TUB2)		N.D.
CREA-DC TPR OL.345^*c*^	Latium, Italy	*Apiospora marii*	PX903890 (ITS), PX894993 (TEF1-α), PX894996 (TUB2)		N.D.
CREA-DC TPR OL.350^*c*^	Latium, Italy	*Apiospora italica*	PX903891 (ITS), PX894994 (TEF1-α), PX894997 (TUB2)		N.D.
CREA-DC TPR OL.427^*d*^	Apulia, Italy	*Neofusicoccum mediterraneum*	OL454501 (ITS), OL539661 (TEF1-α), OL539662 (TUB2)		N.D.
CREA-DC TPR OL.431^*d*^	Apulia, Italy	*Neofusicoccum stellenboschiana*	OP893663 (ITS), OQ091953 (TEF1-α), OQ091957 (TUB2), OQ091961 (RPB2)		N.D.
CREA-DC TPR OL.463^*c*^	Apulia, Italy	*Diplodia olivarum*	PX724635 (ITS), PX753377 (TEF1-α), PX841429 (TUB2)		N.D.
CREA-DC TPR OL.587^*c*^	Apulia, Italy	*Diplodia africana*	PX724680 (ITS), PX753422 (TEF1-α), PX841474 (TUB2)		N.D.
CREA-DC TPR OL.661^*c*^	Apulia, Italy	*Apiospora aurea*	PX724708 (ITS), PX753446 (TEF1-α), PX841498 (TUB2)		N.D.
CREA-DC TPR CP23	Latium, Italy	*Ceratocystis platani*	PZ052643 (ITS), PZ066680 (TEF1-α)		N.D.

^*a*^ N.D., not detected.

^*b*^ Fungal strains and their sequences are reported in references 20, 21.

^*c*^ These fungi have been isolated from olive trees. The sequences are part of an unpublished data set belonging to an ongoing research project.

^*d*^ Fungal strains and their sequences are reported in Brunetti et al. (38) and Manetti et al. (39).

^*e*^ Apart from sequences specified in the footnotes, all sequences have been produced in this study.

^*f*^ FIG, fig tree; OL, olive.

**TABLE 2:** Inclusivity of the real-time PCR method^*d*^

CF isolate code	C*ts* with 500 fg (SD)	C*ts* with 10 fg (SD)	Accession no.
CREA-DC TPR CPC 44183	29.2 (0.06)	34.8 (0.32)	PZ052633 (ITS)^*a*^ PZ066674 (TEF1-α)^*a*^
CPC 44213	29.0 (0.07)	34.4 (0.61)	OQ990098 (ITS)^*b*^ PX994926 (ITS-A)^*c*^ PX994927 (ITS-B)^*c*^ OQ990051 (LSU)^*b*^ OQ989211 (RPB2)^*b*^ OQ989258 (TUB2)^*b*^ OQ989240 (TEF1)^*b*^
CREA-DC TPR CPC 44218	29.9 (0.10)	35.5 (0.77)	PZ052634 (ITS)^*a*^ PZ066675 (TEF1-α)^*a*^
CREA-DC TPR CPC 44222	29.2 (0.07)	34.9 (0.48)	PZ052635 (ITS)^*a*^ PZ066676 (TEF1-α)^*a*^
CREA-DC TPR CPC 44223	30.0 (0.04)	35.6 (1.17)	PZ052636 (ITS)^*a*^ PZ066677 (TEF1-α)^*a*^
CREA-DC TPR CF Sic2	29.9 (0.08)	35.1 (0.04)	PZ052637 (ITS)^*a*^ PZ066678 (TEF1-α)^*a*^
CREA-DC TPR CF Sic5	29.4 (0.10)	34.9 (0.61)	PZ052638 (ITS)^*a*^ PZ066679 (TEF1-α)^*a*^

^*a*^ The remaining sequences were also produced in this study through Sanger sequencing, directly or after cloning into a plasmid vector.

^*b*^ Sequences are reported in references 17, 18.

^*c*^ Sequences ITS-A and ITS-B were obtained through PacBio sequencing and represent the two amplicon sequence variants obtained (ASV) (see also Text S1).

^*d*^ The tested *Ceratocystis ficicola* (CF) isolates were from Sicily (Italy). The mean values of threshold cycles obtained with 500 fg and 10 fg CF gDNA as the template are reported.

We tested 500 pg gDNA for each non-target and target isolate. Target isolates were also tested at 10 fg/PCR. To verify that the non-target gDNAs did not inhibit the amplification, each non-target fungal gDNA was also tested in reactions spiked with 25 fg of CF gDNA. Positive controls consisted of reactions containing 25 fg and 500 fg of CF gDNA; negative controls included no-template reactions. All assays were performed in triplicate.

#### Diagnostic sensitivity and specificity

The diagnostic sensitivity (DSE) was defined as the ability of the method to detect positively the plant samples that are really infected/infested with the target (micro-) organism, namely the capacity not to incur in false-negative detections (a false-negative detection is obtained for samples for which a positive result is expected/assigned). Thus, it is expressed as the ratio between the number of samples positively detected and for which the assigned value is positive (N_TP_ = number of true positives) and the total number of tested samples for which the assigned value is positive (*N*^+^). DSE = N_TP_/*N*^+^ (29).

Diagnostic specificity (DSP) was defined as the ability of the method to detect negatively the plant samples that are not infected/infested with the target (micro-)organism, namely the capacity not to incur in false-positive detections (a false-positive detection is obtained for samples for which a negative result is expected/assigned). Thus, it is expressed as the ratio between the number of samples negatively detected and for which the assigned value is negative (N_TN_ = number of true negatives) and the total number of tested samples for which the assigned value is negative (N^−^). DSP = N_TN_/N^−^ (29).

Validation was conducted using artificially infected samples (obtained as described in the paragraph “Inoculation trials”) and three categories of naturally occurring samples: (i) 10 potted fig trees from a nursery in Sicily exhibiting severe bark cankers and wood discoloration and confirmed to be naturally infected with CF; (ii) trees uninfected with CF and showing wood discoloration caused by other fungi; and (iii) symptomless trees with healthy-looking wood. The latter two categories were trees located in the Latium region and were represented by 20 samples.

To confirm that extracts from CF-negative fig samples did not inhibit amplification, each extract was additionally tested in reactions spiked with 25 fg of CF gDNA. Positive controls consisted of reactions containing 25 fg or 500 fg of CF gDNA, as well as extracts from samples previously confirmed to be CF-infected. Negative controls included no-template reactions and extracts from samples previously confirmed to be CF-uninfected. All assays were performed in triplicate.

### Qualitative validation of the real-time PCR method: applicability for electrophoretic agarose gel analysis

Qualitative validation of the real-time PCR method was performed by analyzing amplification products through electrophoresis on 2% agarose gels. Four categories of PCR amplifications were examined.

- Amplicons generated from reactions containing 500, 10, and 5 fg of CF gDNA. Four technical replicates were analyzed for each DNA quantity, and the experiment was conducted twice.
- Amplification products obtained from DNA extracts of the 10 naturally infected fig samples used in the diagnostic sensitivity validation. Three technical replicates were analyzed; the experiment was performed once.
- Amplification products from DNA extracts of artificially inoculated fig plants used in the diagnostic sensitivity validation. Samples were selected from three plants inoculated with the Japanese CF isolate and three plants inoculated with the Italian (Sicily) CF isolate. For each plant, extracts from two tissue types were amplified and analyzed: (a) xylem from the fully necrotized, heavily colonized area and (b) xylem from the distal, weakly discolored zone where necrosis was incipient. Three technical replicates were analyzed per sample; the experiment was performed once.
- Amplification products from wood DNA extracts from fig trees in the Latium region (Italy) infected with fungi other than CF, both symptomatic and asymptomatic.

Gels from experiment i were stained with ethidium bromide (Merck KGaA, Darmstadt, Germany). Gels from experiments ii, iii, and iv were stained with FluoroVe Nucleic Acid Gel Stain (SMOBIO Technology, Hsinchu City, Taiwan).

## RESULTS

### Real-time PCR: optimization

In the gradient experiments, 62°C was the highest temperature yielding steep fluorescent curves and was thus selected as the definitive annealing/extension temperature. Primer concentration and cycling conditions were deemed appropriate and applied in all subsequent experiments. Agarose gel electrophoresis confirmed the amplification of a single product of the expected size, and sequencing verified that the amplified fragment corresponded precisely to the target CF sequence. Thus, this clearly suggested that primers amplified the target sequence only, ruling out any other non-specific amplification of other non-target sequences from CF gDNA.

No significant fluorescence increase was obtained in the no-template reactions at the selected annealing/extension temperature, and correspondingly, dissociation analysis showed no peaks. This ruled out any tendency of primers to form dimers.

### Efficiency of the amplification process, melting temperature, and impact of the wood matrix

**Fig 1:**
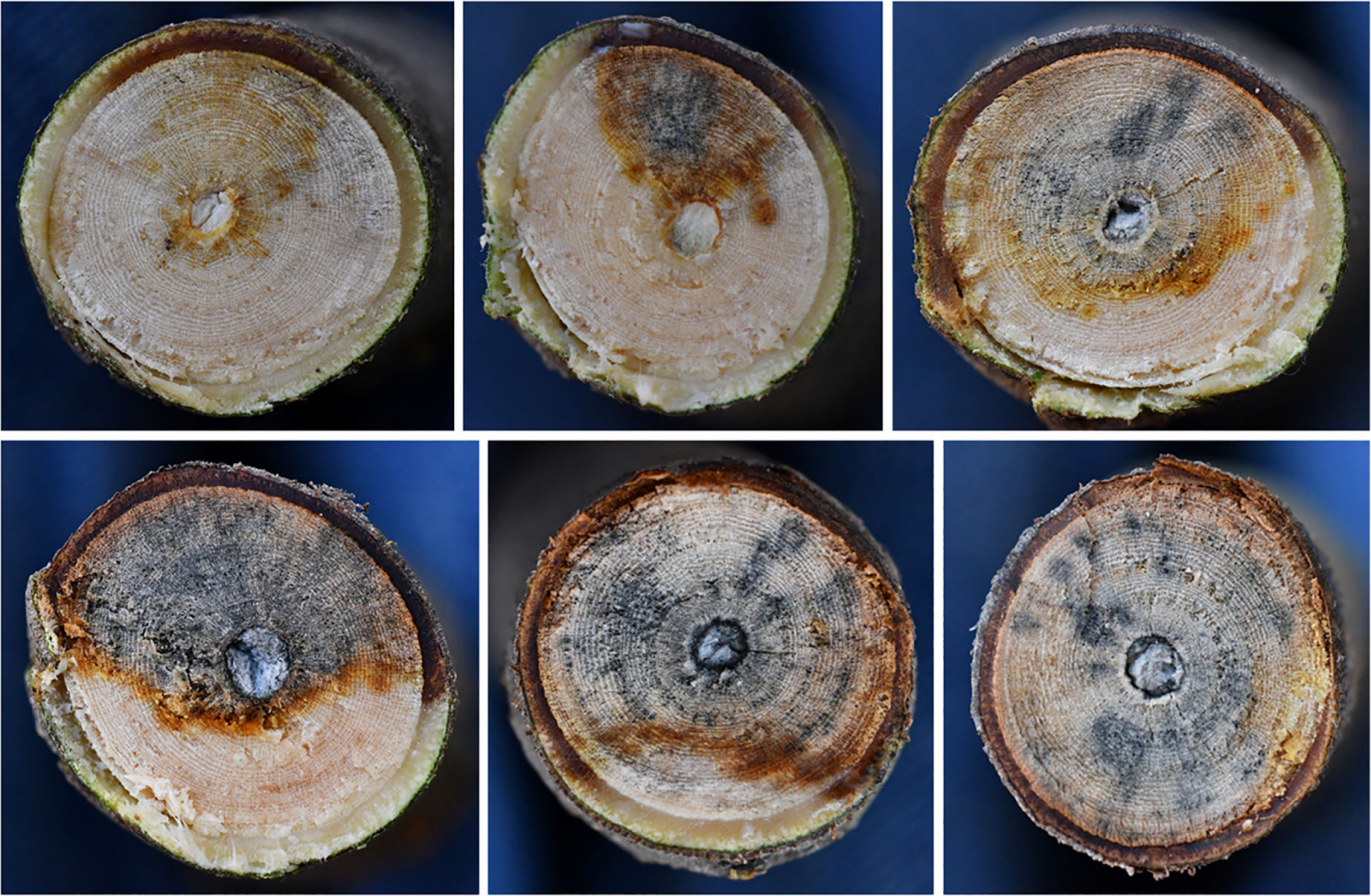
Discoloration pattern in cross sections of branches from a declining fig tree infected with Botryosphaeriaceae, Diaporthaceae, Apiosporaceae, and Bionectriaceae. DNA extracted from this necrotic wood was used to spike real-time PCRs containing *Ceratocystis ficicola* (CF) gDNA to assess the impact of necrotic wood on amplification efficiency and analytical sensitivity of the assay.

To assess the effect of the wood matrix on PCR amplification, we tested 500 fg and 25 fg of CF gDNA either alone or spiked with 1 or 3 µL of a meta DNA extracted from discolored/necrotic wood of two CF-negative fig trees from the Latium region. The two trees were colonized, respectively, by (i) Botryosphaeriaceae and (ii) Botryosphaeriaceae, Diaporthaceae, Apiosporaceae, and Bionectriaceae. Representative symptoms are shown in Fig. 1. Two wood-DNA extraction approaches were also compared.

**Fig 2:**
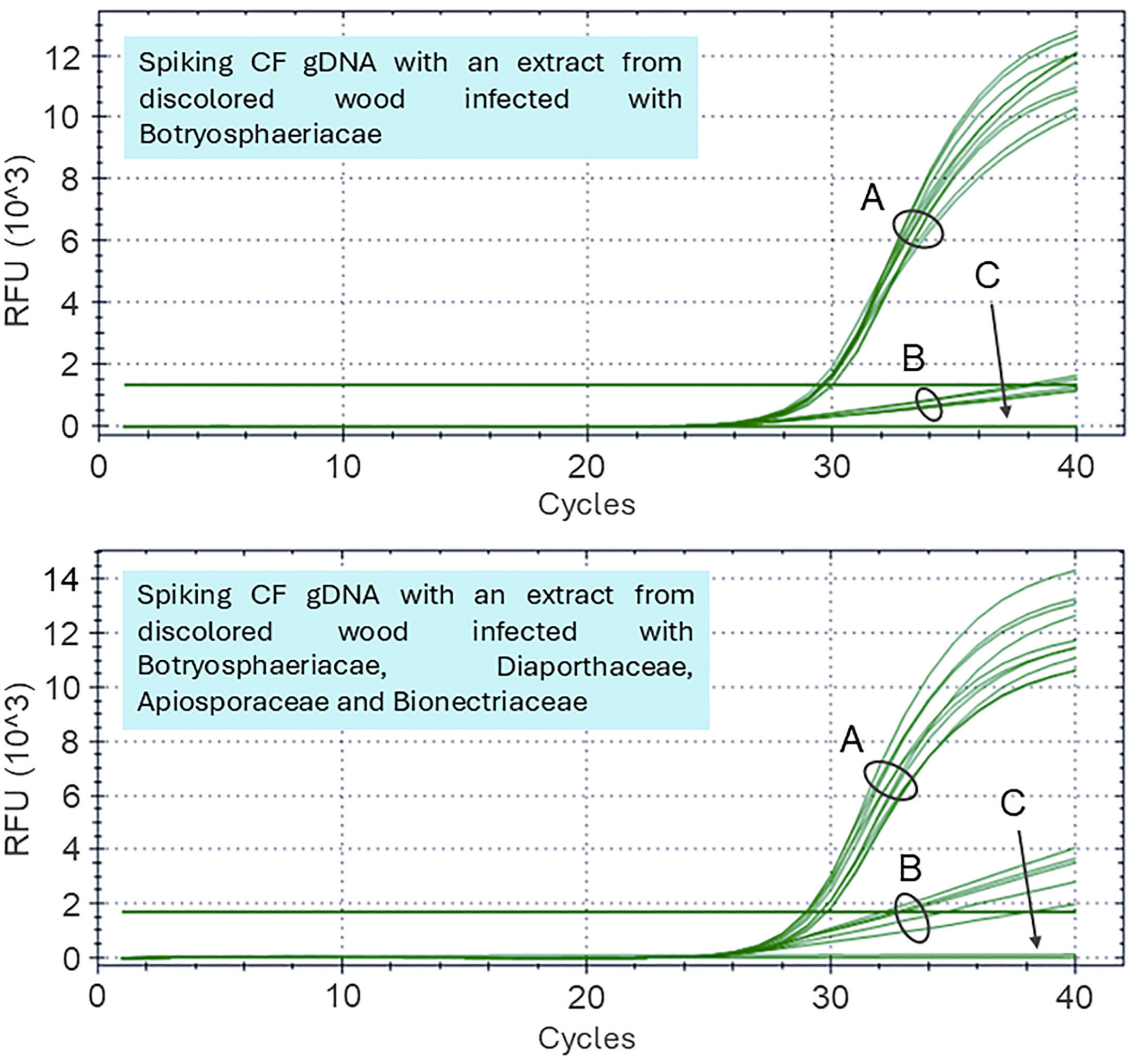
Effect of DNA extracts from discolored/necrotic fig wood, uninfected by *Ceratocystis ficicola* (CF) but infected with other fungi, on the real-time PCR amplification of CF. Extracts were from sample 20-S (the upper real-time PCR run) and 18-S (the lower run) (see also Table 7 for the associated fungal taxa). Bundle A shows signals from (i) positive controls with 500 fg CF gDNA and (ii) 500 fg CF gDNA spiked with 3 μL of necrotic wood extract treated with the DNeasy PowerClean Pro Cleanup Kit for inhibitor removal. Bundle B shows reactions with 500 fg CF gDNA spiked with 3 μL of untreated necrotic wood extract. “C” denotes no-template controls spiked with treated or untreated necrotic wood extract that yielded no signal. For the sake of immediacy, the graph does not depict a part of the whole experiment, which also investigated the effects of the addition of healthy wood extract, 1 μL of wood extract (both necrotic and healthy), and the amplification of 25 fg CF gDNA (see the text and Table S2 for details on the complete experiment).

Across all conditions, spiking reactions with necrotic-wood extracts markedly increased Ct values and frequently caused a collapse of fluorescence curves relative to non-spiked reactions (Fig. 2). Inhibition was most pronounced with 3 µL of extract, whereas 1 µL produced only minor effects. Notably, treatment of the necrotic-wood extracts with the DNeasy PowerClean Pro Cleanup Kit effectively eliminated inhibition: all spiked positive reactions showed Ct values and fluorescence profiles comparable to non-spiked controls (Fig. 2; Table S2). In contrast, spiking CF gDNA with extracts from asymptomatic wood not subjected to inhibitor removal yielded amplification curves indistinguishable from those of non-spiked controls (Table S2). These results indicate that the observed inhibition is specifically associated with components enriched in necrotic wood.

The findings described above guided the systematic investigation of real-time PCR efficiency using dilution-series amplification and standard curve analysis. For mycelial DNA and healthy-looking wood, we employed the simplest extraction workflow (DNeasy Plant Maxi/Mini Kit) to generate standard curves with CF gDNA alone, and CF gDNA spiked with 2 µL of extract from healthy-looking wood. For necrotic wood—both fresh and dried—the DNeasy PowerClean Pro Cleanup Kit was coupled with the DNeasy Plant Maxi kit prior to standard-curve generation. In total, four types of standard curves were evaluated.

**Fig 3:**
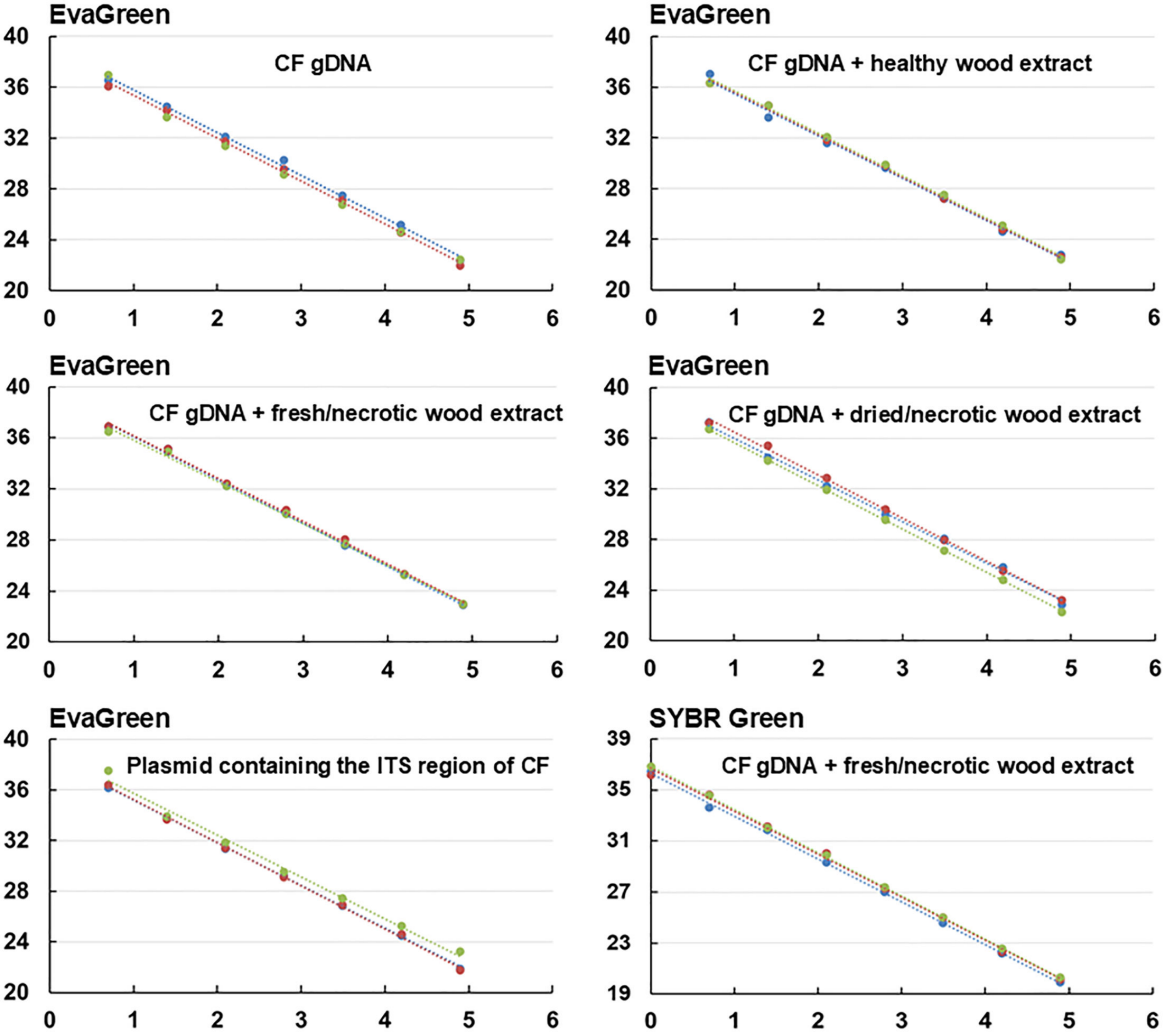
Amplification efficiency and selectivity assessed by standard curves generated from fivefold serial dilutions of *Ceratocystis ficicola* (CF) gDNA (78.12 pg to 5 fg for EvaGreen and 1 fg for SYBR Green) and of a plasmid containing the complete ITS region of *Ceratocystis ficicola* (CF) (3.1 pg to 200 ag). Assays were performed using CF gDNA alone and spiked with different extracts of fig wood matrices to evaluate their impact on amplification efficiency (selectivity; the dried/necrotic and fresh/necrotic wood extracts were from samples 19S and 20S, respectively, see Table 7). Each experiment type was conducted in triplicate, with three technical replicates per dilution point. Y-axis: Ct values; X-axis: log of starting CF gDNA quantity or CF ITS region-containing plasmid quantity. See also Table 3 for values of performance parameters.

Across all assay types, PCR efficiency was consistently high and reproducible, ranging from 95.7% to 102.6% (Table 3; Fig. 3). Linearity and slope values similarly approached best fitting values (Table 3; Fig. 3). Dilution series prepared in the presence of different wood extracts also enabled a preliminary estimation of assay sensitivity. Five femtograms of CF gDNA per reaction were reliably detected in all four standard-curve formats (12 independent experiments), with mean Ct values between 36.1 and 37.3. The lowest dilution points tested—1 fg and 200 ag per reaction—were inconsistently detected, depending on the experiment.

**TABLE 3:** Standard curves evaluating the performance of the EvaGreen- and SYBR Green-based real-time PCR assay for detection of *Ceratocystis ficicola* (CF)^*d*^

Dye	Standard curve	*E*^*a*^ (%)	*R* ^2^ ^*b*^	Slope^*c*^	Intercept
EvaGreen	I—CF gDNA	98.3	0.991	−3.36	39.2
	II—CF gDNA	97.8	0.995	−3.38	38.8
	III—CF gDNA	97.7	0.994	−3.38	38.7
EvaGreen	I—CF gDNA + HW fig wood DNA	99.5	0.990	−3.33	38.8
	II—CF gDNA + HW fig wood DNA	99.3	0.994	−3.34	38.9
	III—CF gDNA + HW fig wood DNA	99.2	0.994	−3.34	39.0
EvaGreen	I—CF gDNA + NW fig wood DNA	97.5	0.994	−3.38	39.5
	II—CF gDNA + NW fig wood DNA	98.7	0.994	−3.35	39.5
	III—CF gDNA + NW fig wood DNA	101.5	0.994	−3.29	39.1
EvaGreen	I—CF gDNA + NDW fig wood DNA	102.6	0.990	−3.26	39.2
	II—CF gDNA + NDW fig wood DNA	96.7	0.985	−3.40	39.9
	III—CF gDNA + NDW fig wood DNA	95.7	0.990	−3.43	39.1
EvaGreen	I—ITS target-containing plasmid (circular)	98.2	0.997	−3.37	43.9
	II—ITS target-containing plasmid (circular)	96.7	0.995	−3.40	44.1
	III—ITS target-containing plasmid (circular)	100.8	0.990	−3.30	44.3
EvaGreen	I—ITS target-containing plasmid (linear)	89.5	0.995	−3.60	45.2
	II—ITS target-containing plasmid (linear)	94.8	0.997	−3.45	44.6
	III—ITS target-containing plasmid (linear)	90.7	0.996	−3.57	44.5
SYBR Green	I—CF gDNA + NW fig wood DNA	97.8	0.999	−3.38	36.3
	II—CF gDNA + NW fig wood DNA	98.3	0.992	−3.36	36.7
	III—CF gDNA + NW fig wood DNA	97.0	0.997	−3.39	36.9

^*a*^ *E* = PCR efficiency, 100% is the maximum theoretical value, which means perfect doubling of molecules at each cycle.

^*b*^ *R*^2^ is a measure of data linearity among technical replicates of the same and the different serial dilutions; one is the best fit.

^*c*^ The slope is the angular coefficient (m) of the equation for the standard curve (*y* = *mx* + *b*); −3.32 is the best fit.

^*d*^ Curves were generated from seven/eight 5-fold serial dilutions of CF gDNA (78.12 pg to 5 or 1 fg for EvaGreen and SYBR Green, respectively). Reactions were tested either alone or spiked with 2 μL of fig wood DNA extracts obtained from healthy-looking tissues (HW) or from discolored/necrotic tissues, either fresh (NW) or dried (NDW) infected with fungi other than CF. Curves obtained with amplifications of the ITS region-containing plasmid—both in the circular and linearized form—were based on seven serial dilutions from 3.1 pg to 8 ag plasmid DNA.

The melting analyses performed following all serial dilution amplifications, using a temperature ramp of 0.1°C, revealed a consistent melting-temperature range of 72.1°C to 72.6°C. The lowest melting temperatures were associated with the lowest template concentrations (25 and 5 fg per PCR).

**Fig 4:**
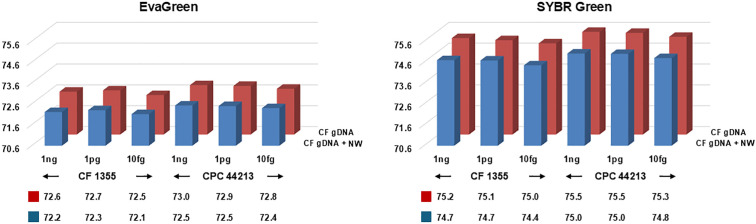
Determination of the melting temperature of the target amplicon amplified in EvaGreen and in SYBR Green and as a function of (i) the *Ceratocystis ficicola* (CF) isolates CPC 44213 (from Sicily, Italy) and CF 1355 (from Japan), (ii) the amount of fungal gDNA, and (iii) the presence of necrotic wood extract (NW).

A dedicated experiment was conducted to compare melting temperatures obtained with EvaGreen and SYBR Green and to evaluate the effects of target template quantity, wood-extract supplementation, and isolate identity. In both EvaGreen and SYBR Green assays, the Japanese isolate consistently displayed a slightly lower melting-temperature range than the Sicilian isolate. The addition of wood extract to the PCR mixture and reductions in template DNA similarly produced small decreases in melting temperature. These differences were detected with full repeatability and reproducibility. Overall melting temperature of the target amplicon varied in the range 72.1–73 with EvaGreen assay and 74.4–75.5 with SYBR Green assay. Across all matched treatments, melting temperatures generated with SYBR Green were consistently 2.3°C–2.6°C higher than those obtained with EvaGreen. See Fig. 4 for details.

Standard curves generated from dilution series of the circular ITS region-containing plasmid yielded near-optimal amplification efficiencies (96.7%–100.8%). In contrast, standard curves obtained using the linearized plasmid showed reduced efficiencies (89.5%–94.8%) (Table 3; Fig. 3). Despite these differences, detection Cts were comparable between circular and linearized plasmid templates, respectively, with values of 31.5 and 31.7 at 5 fg, 33.8 and 34.1 at 1 fg, and 36.7 for both templates at 200 ag (values are the mean from nine replicates). The lowest plasmid DNA amount consistently detected with full repeatability and reproducibility was 200 ag per reaction.

Dilution series spiked with fresh necrotic wood extract were also amplified using the SYBR Green-based supermix. As observed with the EvaGreen-based assays, PCR efficiency remained high and reproducible, ranging from 97% to 98.3% (Table 3; Fig. 3). Linearity and slope values similarly approached optimal fits (Table 3; Fig. 3). Across the three independent experiments, 1 fg of CF gDNA per reaction was consistently detected, with mean Ct values of 36.1, 36.8, and 37.1. The lowest concentration tested (200 ag per reaction) was detected inconsistently and varied among experiments.

### Analytical sensitivity

**Fig 5:**
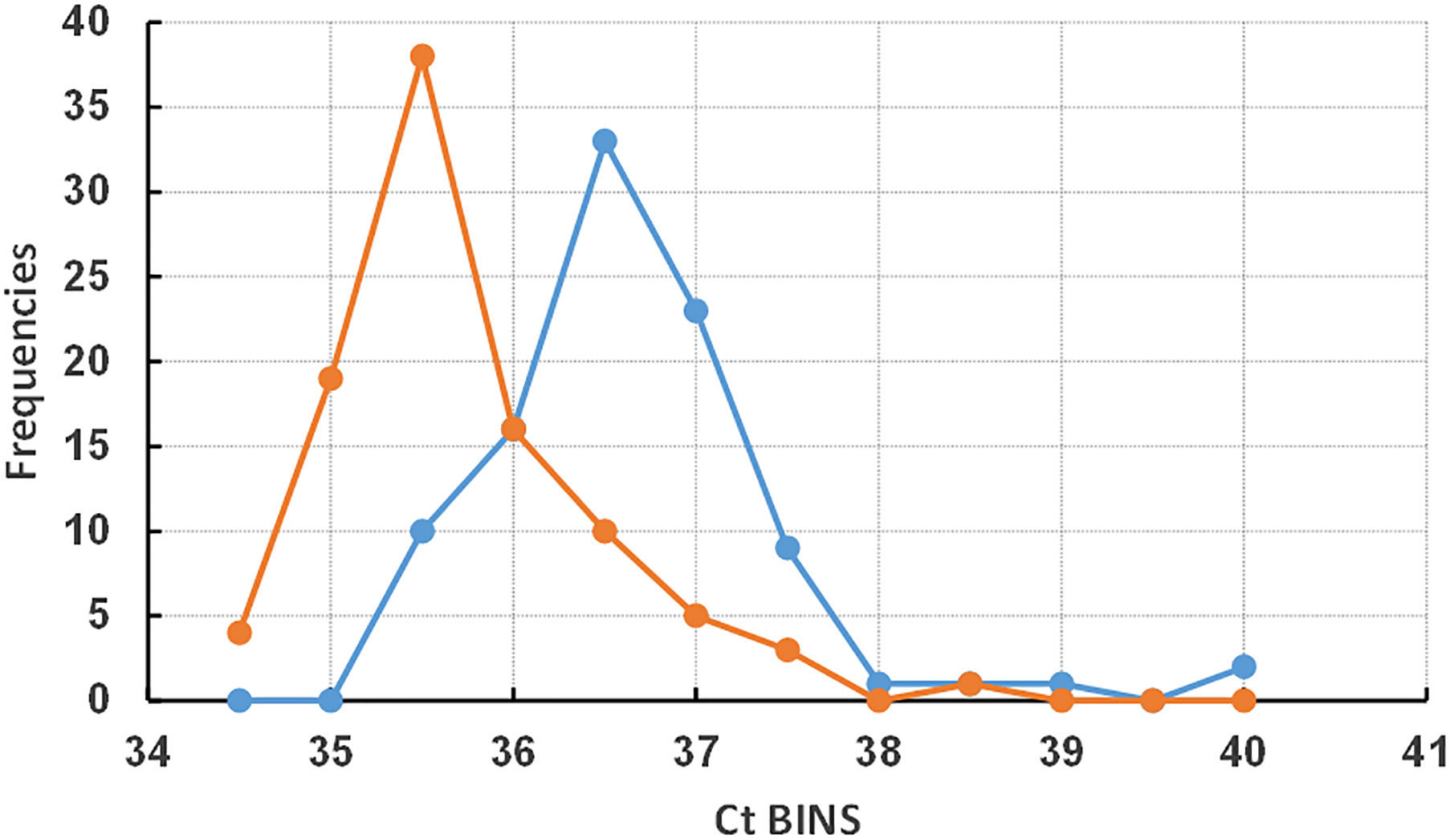
Validation of analytical sensitivity of the EvaGreen-based assay. Frequency distribution of Ct values at the two tested LoD: 5 fg (blue curve) and 10 fg (orange curve) per PCR. Distributions include all 96 Ct values for each LoD, obtained from *Ceratocystis ficicola* (CF) gDNA alone and spiked with various wood extracts. The x-axis BINs represent intervals of 0.5 Ct units, with each BIN labeled by its upper boundary (e.g., 35 represents the 34.5–35 range).

Dilution-series experiments with EvaGreen-based supermix showed that 5 fg of CF gDNA were detectable with full repeatability and reproducibility. However, most Ct values for 5 fg clustered within Ct 36–37, a range considered borderline for reliable detection due to the increased risk of overlap with low-level contamination signals. Consequently, we decided that the operational LoD had to be set to correspond to Ct values of ~35–36. Based on this threshold, subsequent DQP-ASe validation experiments—conducted with a high number of replicates—evaluated both 5 fg and 10 fg of CF gDNA as candidate LoD values. Both DNA quantities were consistently detected across all conditions, irrespective of whether CF gDNA from pure culture was tested alone or spiked with wood extracts. For 5 fg, mean Ct values of the various experiments ranged from 36.3 to 36.6, with individual replicates spanning 35.0–39.6. In contrast, 10 fg produced earlier mean Ct values–35.2–36.1—with an earlier range of variability for individual replicates, 34.3–38.3. Detailed Ct means for each experiment are provided in Table 4, and the frequency distribution of individual Ct values is shown in Fig. 5.

**TABLE 4:** Analytical sensitivity of the EvaGreen-based real-time PCR assay^*a*^

Treatment	gDNA CF		gDNA CF + DNA healthy-looking wood		gDNA CF + DNA fresh necrotic wood		gDNA CF + DNA dried necrotic wood		Amplicon target-containing plasmid	
Limit of detection (LoD)	5fg	10fg	5fg	10fg	5fg	10fg	5fg	10fg	200 ag	500 ag
I exp.	36.5 (0.8)	35.1 (0.4)	36.4 (0.7)	35.0 (0.3)	36.6 (1.3)	35.7 (0.6)	35.6 (0.4)	34.9 (0.4)	36.8 (0.4)	35.3 (0.4)
II exp.	36.6 (0.3)	35.0 (0.3)	36.3 (0.6)	35.4 (0.5)	36.4 (0.6)	35.9 (0.8)	36.4 (0.7)	35.3 (0.5)	36.8 (0.4)	35.6 (0.3)
III exp.	36.0 (0.5)	35.5 (0.8)	36.2 (0.3)	36.0 (0.6)	36.8^*b*^ (1.2)	36.5 (0.8)	37.0 (0.9)	35.3 (0.4)	36.7 (0.5)	35.6 (0.3)
Overall	36.4 (0.6)	35.2 (0.5)	36.3 (0.5)	35.5 (0.6)	36.6 (1.1)	36.1 (0.8)	36.4 (1.0)	35.2 (0.4)	36.8 (0.4)	35.5 (0.4)

^*a*^ Mean threshold cycles (C*t*) ± SD (in parentheses) for detection of 5 and 10 fg *Ceratocystis ficicola* (CF) gDNA, either alone or spiked with DNA extracts from healthy-looking or necrotic (fresh and dried) fig wood. Each DNA quantity, treatment, and experiment included eight technical replicates. Gray shading indicates the operational limit of detection (LoD).

^*b*^ One replicate of eight was not detected (NA).

Based on these results, 10 fg was defined as the operational LoD for the EvaGreen-based assay.

For experiments using the PCR target–containing plasmid, only the circular form was used for analytical sensitivity assays, based on its near-optimal amplification efficiency and slightly lower Ct values than the linearized form. Experiments demonstrated that both 500 ag and 200 ag of plasmid DNA were detected with full repeatability and reproducibility. Amplification of 500 ag yielded a mean Ct of 35.5, comparable to that obtained with 10 fg of CF gDNA, whereas 200 ag produced a mean Ct of 36.8, similar to the detection of 5 fg of CF gDNA (Table 4). These data indicate that 500 ag of recombinant plasmid DNA contains approximately the same number of ITS copies as 10 fg of CF gDNA, the established LoD. Based on the molecular weight of the recombinant plasmid, 500 ag corresponded to ~123 ITS copies, representing the EvaGreen-established LoD in a 30 µL reaction—equivalent to approximately 4 ITS copies per microliter of PCR.

Validation of DQP-ASe with the SYBR Green-based supermix showed that this dye yielded an LoD even lower than that achieved with the EvaGreen-based formulation. In fact, both 5 fg and 3 fg of CF gDNA were consistently detected with full repeatability and reproducibility in the presence of wood extract. Specifically for 3 fg, mean Ct values ranged from 35.4 to 35.6, with individual replicates spanning 34.6–37.1. Five femtograms produced earlier mean Ct values—34.1–34.9—with an earlier range of variability for individual replicates, 33.6–35.4 (Table 5). Since we determined that the operational LoD should fall within the 35–36 Ct interval, 3 fg was identified as the operational LoD for the SYBR Green–based assay. Based on our estimate that 10 fg of CF gDNA contains approximately 123 ITS copies, 3 fg corresponds to ~37 ITS copies (123/10 × 3) (i.e., roughly 1 ITS copy per microliter of PCR mixture), which therefore represents the estimated LoD of the SYBR Green assay.

**TABLE 5:** Analytical sensitivity of the SYBR Green-based real-time PCR assay^*a*^

Treatment	gDNA CF + DNA fresh necrotic wood	
Limit of detection (LoD)	3 fg	5 fg
I exp.	35.5 (0.72)	34.9 (0.51)
II exp.	35.6 (0.69)	34.4 (0.32)
III exp.	35.4 (0.66)	34.1 (0.55)
**Overall**	**35.5** (**0.66**)	**34.4** (**0.56**)

^*a*^ Mean threshold cycles (C*t*) ± SD (in parentheses) for detection of 3 and 5 fg *Ceratocystis ficicola* (CF) gDNA spiked with fresh necrotic fig wood extract. Each DNA quantity and experiment included eight technical replicates. Gray shading indicates the operational limit of detection (LoD).

### PacBio sequencing

After PacBio sequencing, 55.945 (CF isolate C1355) and 60.577 (CF isolate CPC 44213) effective circular consensus sequencing (CCS) were obtained. Overall, two ASVs classified as *C. ficicola* ITS were detected. Both ASVs were present in the two analyzed isolates; however, in CPC 44213, ASV-A was predominant compared with ASV-B (58.303 vs 301 CCS), whereas in C1355, the opposite was observed (15 vs 55.475 CCS). The two ASVs showed an identity of 96.6% (length of 664/681 bp) and 19 gaps. The coexistence of two ITS ASVs clearly suggests the presence of two allelic variants, which may account for the inconsistent or ambiguous results occasionally obtained by direct Sanger sequencing. The primers used in this study for real-time PCR spanned regions conserved between the two ASVs. See Text S1 for details.

### Analytical specificity: exclusivity and inclusivity

Regarding exclusivity, none of the 50 non-target fungal isolates produced detectable fluorescence signals, and no melt peaks were observed after dissociation analysis (Table 1). Reactions containing mixtures of non-target fungal gDNA and CF gDNA generated clear amplification curves and a melt peak identical to that of CF, indicating that non-target gDNAs did not interfere with amplification and confirming that the absence of signals in the exclusivity tests reflected true non-amplification.

Regarding inclusivity, all eight CF isolates were successfully detected at both 500 fg and 10 fg of gDNA per reaction. Mean Ct values for 10 fg ranged from 34.5 to 35.6 (Table 2), consistent with those obtained for 10 fg in the analytical sensitivity experiments using the Japanese isolate (Table 4).

### Symptom expression in artificially infected plants and diagnostic performance in collected samples

Pathogenicity trials were conducted on fig trees using two CF isolates (from Japan and Italy), which represent the target of the real-time PCR assay, and some Botryosphaeriaceae fungi isolated from fig, representing non-target taxa.

In control trees inoculated with sterile PDA, no bark necrosis developed, and inoculation sites had fully healed by the end of the experiment. Aside from wound-associated discoloration, no additional streaking was observed. Three wood aliquots, each from a different plant, were collected from tissues immediately behind the inoculation point. All three aliquots yielded negative PCR signals.

#### Diagnostic sensitivity

**Fig 6:**
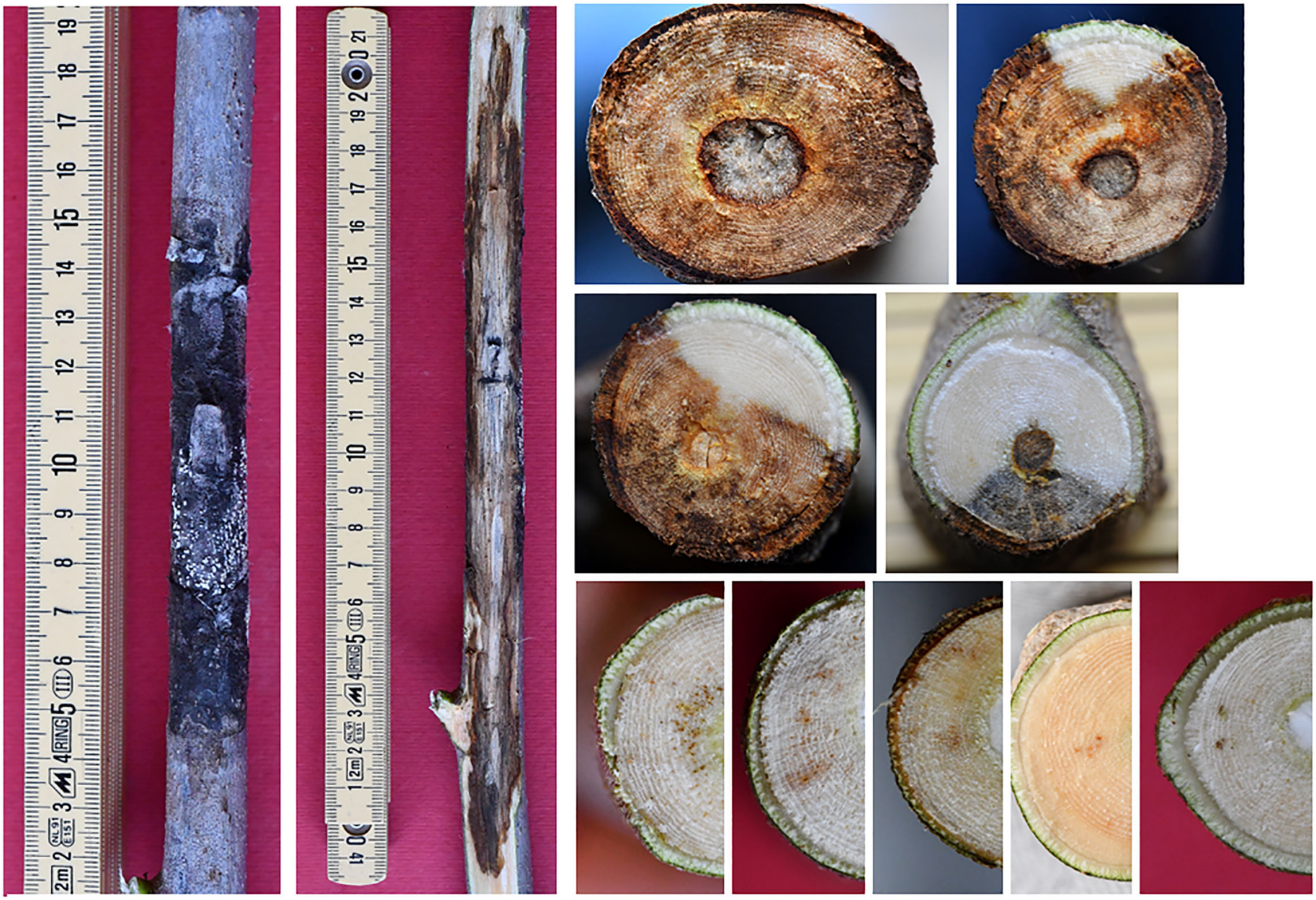
Necrotic symptoms induced by inoculation of fig trees with *Ceratocystis ficicola* (CF). Bark cankers and wood discoloration/necrosis are shown in longitudinal and cross-sections of the stem. In cross-section, necrosis exhibits a wedge-shaped pattern where the disease is fully established, while at the advancing front, along the stem, it appears as scattered reddish-brown dot-like spots that are increasingly sparse and faint. Real-time PCR diagnostic sensitivity was validated under both symptom conditions.

As expected, both CF isolates exhibited markedly higher virulence than the Botryosphaeriaceae. Four of five trees inoculated with the Japanese isolate and three of five inoculated with the Italian-Sicilian isolate wilted and died within three months (the time course of the trial). All CF-inoculated trees developed extensive bark and wood necrosis radiating both acropetally and basipetally from the inoculation point. Bark necrosis girdled most stems completely, and no callus formation was observed at lesion margins. In the wood, a dark longitudinal and transverse discoloration was consistently present. In longitudinal section, the necrotic streak consisted of two components: (i) a primary zone directly expanding from the inoculation site with uniform, intense discoloration and (ii) distal acropetal and basipetal extensions composed of sparse, thread-like streaks. Correspondingly, cross-sections showed a wedge- to arch-shaped lesion penetrating 70%–100% of the stem diameter in the primary zone, while a scattered dot-like discoloration was present in the distal zone (Fig. 6). CF was successfully reisolated from all symptomatic tissues.

**Fig 7:**
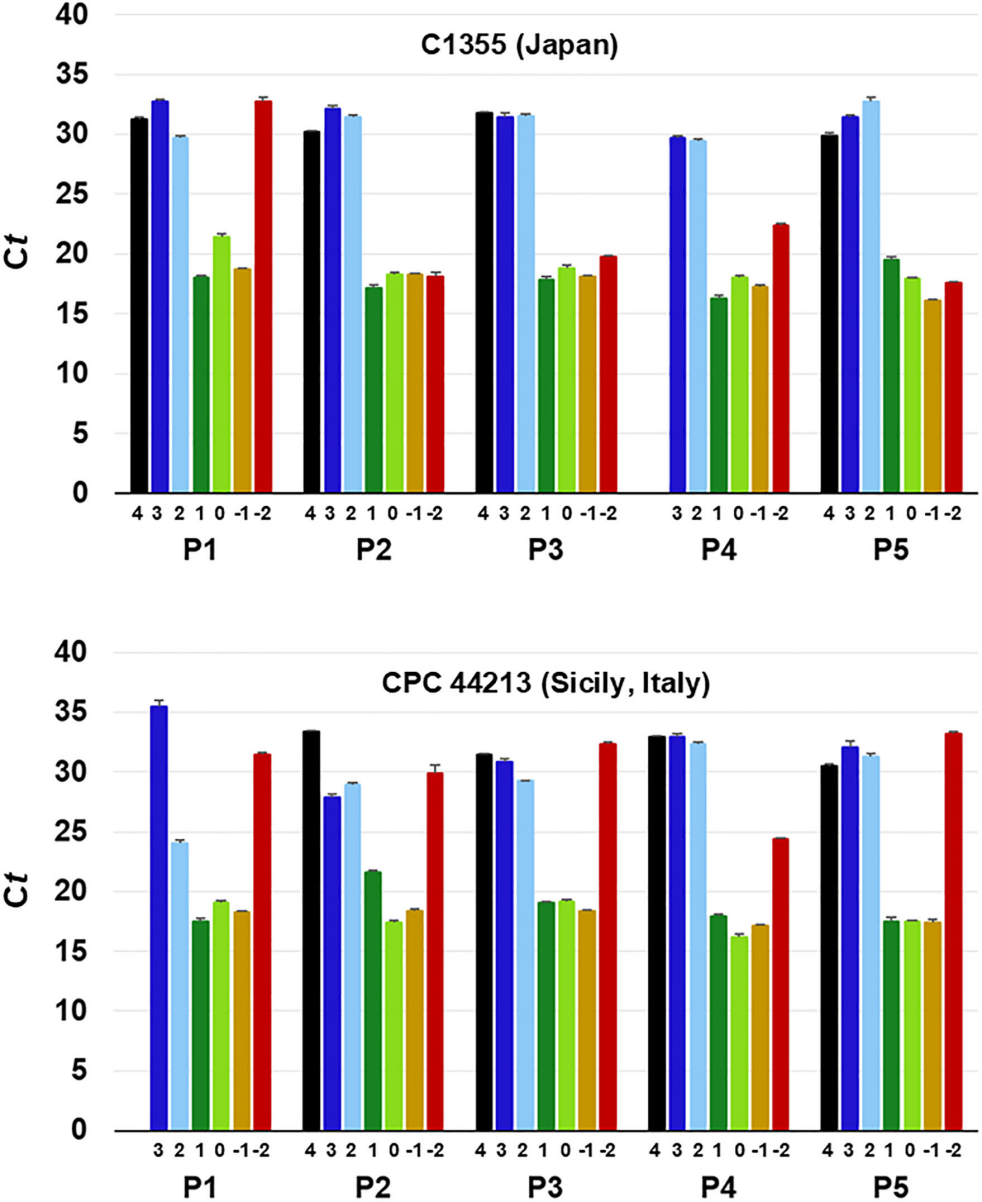
Diagnostic sensitivity validated on samples from fig trees artificially inoculated with *Ceratocystis ficicola* (CF) isolates from Japan (C1355) and Italy (Sicily) (CPC 44213). Each isolate was inoculated onto five plants (P1–P5). From each plant, six to seven aliquots were collected from necrotic lesions at the inoculation point (0), upper and lower margins of the homogeneous necrotic streak component (1 upward and –1 downward), and multiple points along the thread-like necrotic streak component spaced 3 cm apart (2, 3, 4 upward; –2 downward). Error bars represent the standard deviation of three technical replicates.

A total of 68 wood aliquots were collected from both components of the necrotic streaks, representing varying distances from the inoculation point and different lesion intensities. This sampling strategy allowed us to challenge the assay under contrasting diagnostic conditions: sparse, thread-like necrosis (limited fungal colonization) vs dense, continuous necrosis (extensive colonization), while also accounting for potential PCR inhibition linked to necrotic tissues, likely more pronounced in the primary than in the distal zone. All 68 aliquots yielded positive PCR signals, giving the best-fitting diagnostic sensitivity, DSE = N_TP_/*N*^+^ = 68/68 = 1.0. Notably, amplification curves were of high quality even though we used 2 µL of untreated wood extract, indicating negligible inhibition. Across all CF-infected trees, Ct values were consistently lower in aliquots from fully necrotic tissue than in those from thread-like distal regions (Fig. 7).

#### Diagnostic specificity

Inoculation with Botryosphaeriaceae fungi resulted in variable disease outcomes depending on the species, but in all cases, the symptoms were markedly less severe than those caused by CF. *Neofusicoccum parvum* induced pronounced dark necrotic lesions in both bark and wood, progressing acropetally and basipetally, and representing the most severe outcome among the Botryosphaeriaceae tested. In the bark, the girdling index (ratio between the tangential spread of necrosis and the stem circumference) ranged from 0.37 to 0.59, with a slight callus reaction observed at lesion margins. In the wood, necrotic streaks exhibited a two-component pattern similar to that described for CF-inoculated trees, but radial penetration was limited to ~3 mm (≈11% of stem diameter). All trees inoculated with *N. parvum* remained viable throughout the observation period (3 months).

**Fig 8:**
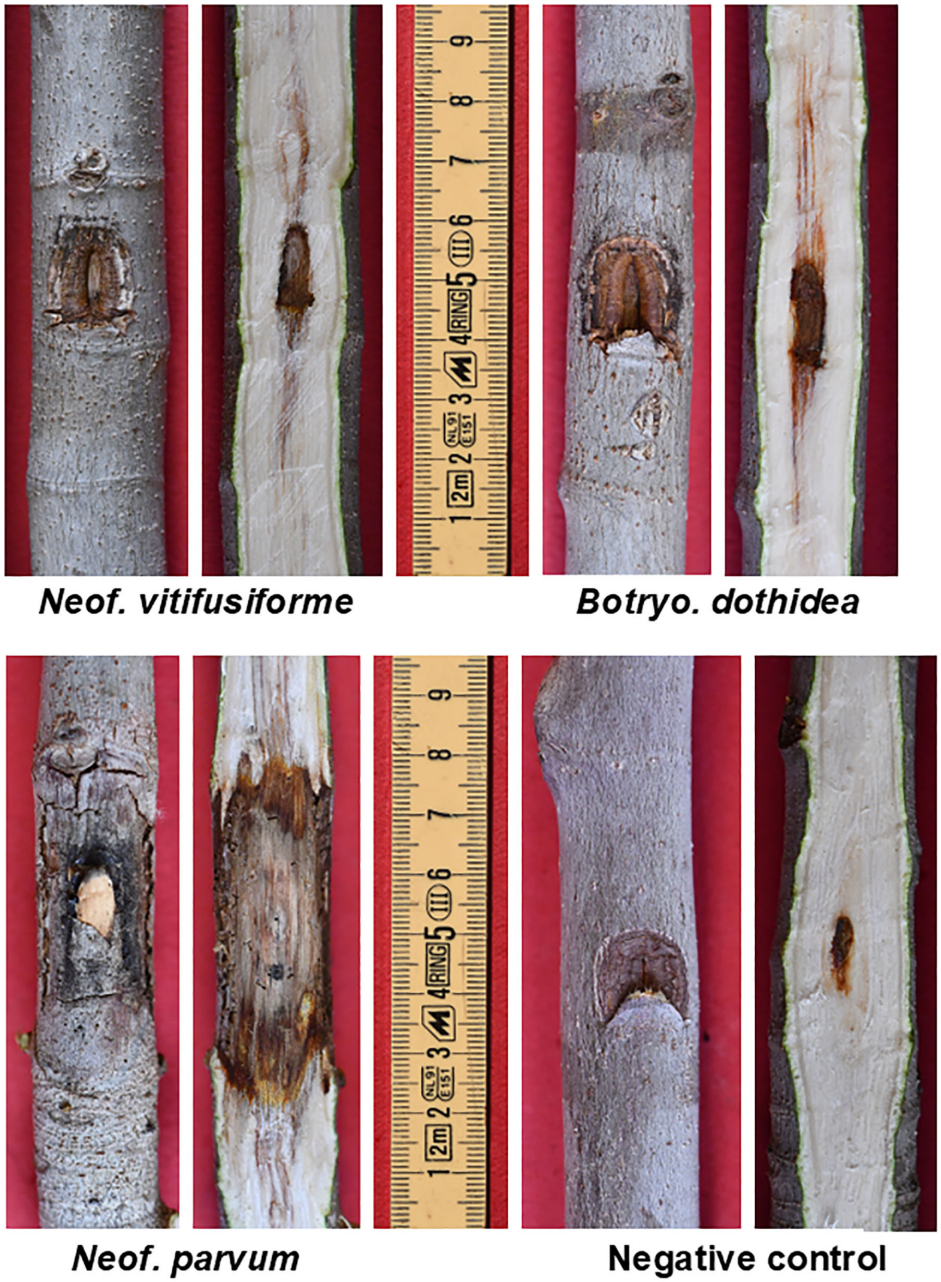
Outcome of inoculation of fig trees with *Botryosphaeriaceae* fungi. *Neof. = Neofusicoccum, Botryo. = Botryosphaeria*.

In contrast, inoculation with *B. dothidea* and *N. vitifusiforme* resulted in very mild symptoms, indicating substantially lower virulence than CF and *N. parvum*. No bark necrosis was observed, and strong callus formation nearly completely healed the inoculation wounds by the end of the trial. In the wood, only small discoloration streaks extended from the inoculation site, slightly longer in *B. dothidea*-inoculated trees than in those inoculated with *N. vitifusiforme*, with no significant radial penetration into internal vascular tissues. Representative outcomes of Botryosphaeriaceae inoculations are shown in Fig. 8.

**Fig 9:**
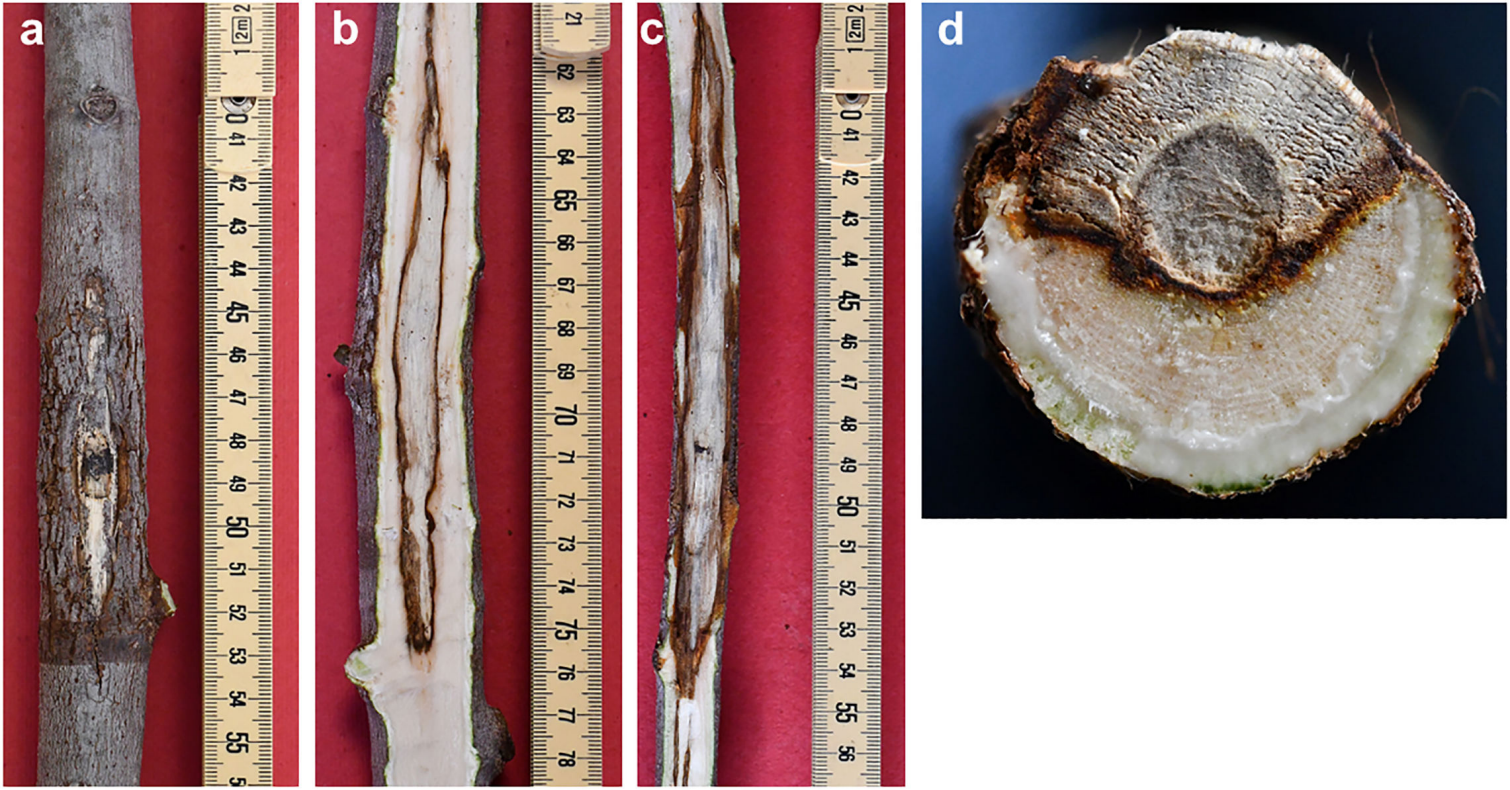
Symptom expression following inoculation of fig trees with *Ceratocystis platani*. (**a**) External bark canker with moderate callus formation at the lesion margin. (**b and c**) Longitudinal sections showing necrotic streaks in the wood beneath the bark canker. (**d**) Cross-section depicting radial necrosis progression forming a wedge-shaped canker.

Inoculation of *C. platani* on fig trees unexpectedly produced pronounced necrotic streaks in both bark and wood. In the bark, cankers initially developed without callus formation at lesion margins, although moderate callus production was evident by the end of the observation period. In the wood, necrosis extended longitudinally from 13.8 to 30.1 cm (μ = 15.7 cm, SD = 9.9 cm) and radially across 42%–61% of the stem diameter, forming wedge-shaped cankers that progressed from the bark to the pith. Despite these evident symptoms, all *C. platani*-inoculated trees remained viable throughout the experiment (Fig. 9).

In contrast, inoculation of the Italian-Sicilian CF isolate on plane trees induced a rapid callus response that completely healed the inoculation site by the end of the observation period. In the wood, a limited orange-brown discoloration extended only a few millimeters beyond the wound, likely reflecting an initial fungal colonization that was rapidly halted and thus unsuccessful.

A graphical representation of virulence levels across all fungal isolates, in terms of loss of viability, necrosis spread, and girdling index, is provided in Fig. S1.

All Botryosphaeriaceae species were successfully reisolated from the discolored wood of the inoculated trees. In contrast, neither CF nor *C. platani* was recovered from heterologous host-pathogen combinations. This was consistent with the plane tree-CF interaction, which revealed an incompatible system characterized by a highly resistant phenotype. For the fig tree-*C. platani* combination, the plant-pathogen pair was fully compatible, as evidenced by the development of severe symptoms; thus, the failure to reisolate the pathogen likely reflects false-negative results, which are commonly observed when detecting *C. platani* through culture-based isolation (28, 40).

A total of 16 wood aliquots were collected from Botryosphaeriaceae- and *C. platani*-inoculated fig trees (one per inoculated plant). All 16 aliquots tested negative, as expected, yielding a diagnostic specificity of DSP = N_TN_/N ^−^ = 16/16 = 1, representing the optimal value.

### Diagnostic performance in natural samples

The 10 potted fig trees obtained from a commercial nursery in Sicily, all exhibiting severe cankers and wilt, were confirmed to be infected with CF by isolating the pathogen on PDA plates and Sanger sequencing.

**Fig 10:**
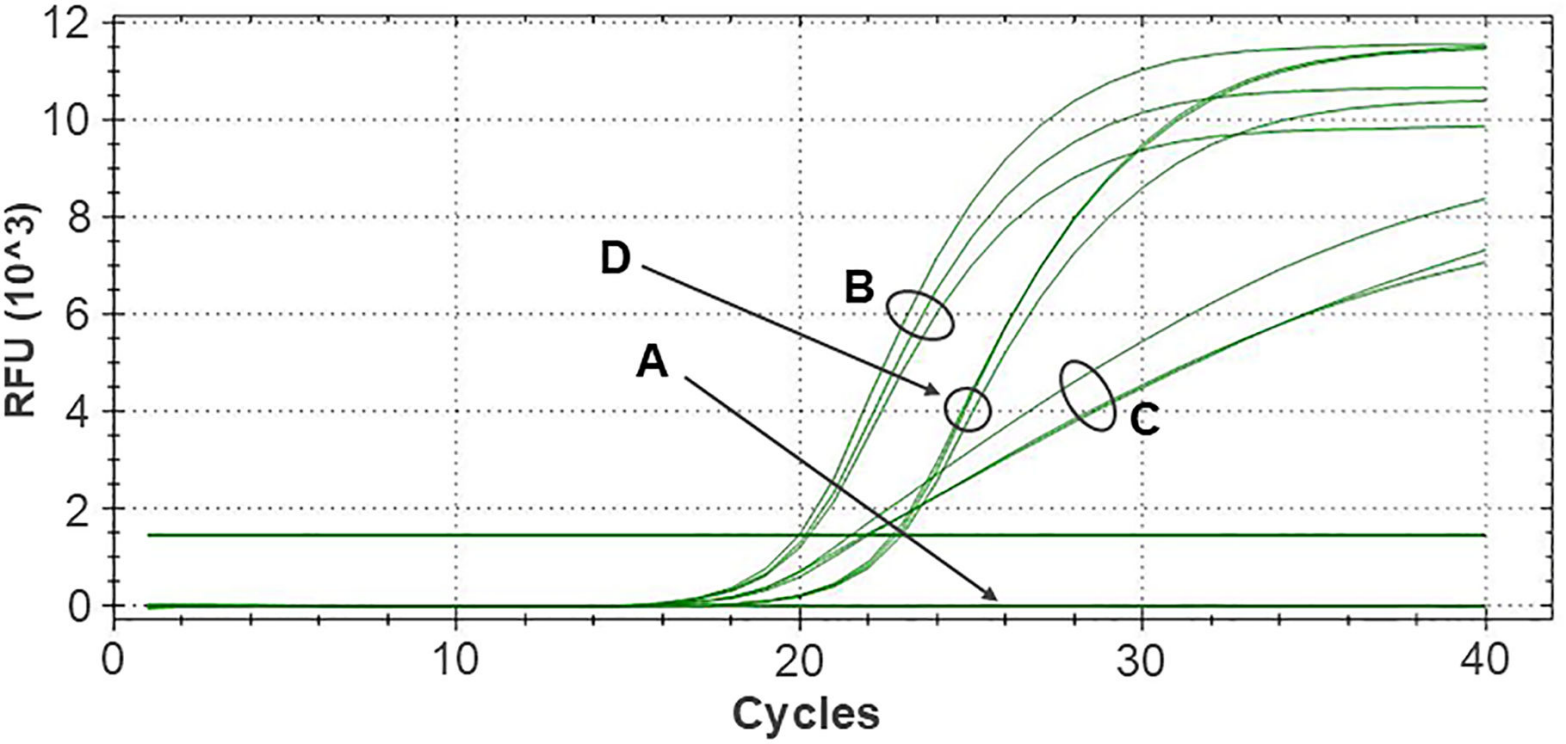
Detection signals from a sample naturally infected with *Ceratocystis ficicola* (CF)and containing substantial PCR inhibitors. In A, results of detection of (i) no-template controls and (ii) reactions with 2 μL of untreated extract, spiked or unspiked with 500 fg CF gDNA. Bundle B shows reactions with 2 μL of extract treated with the inhibitor removal kit. Bundle C shows reactions with 1 μL of untreated extract, and bundle D shows reactions with 2 μL of 1:10 diluted extract. It should be noted that reducing template volume, diluting the extract, and using the cleanup kit all help overcome amplification inhibition; however, only treatment with the inhibitor removal kit fully restores diagnostic signals at the earliest Ct.

All wood samples were tested by real-time PCR, which confirmed the infections. Specifically, detection using extracts obtained solely with the DNeasy Plant Mini Kit yielded high-quality fluorescent signals for five samples; three samples produced collapsed but still interpretable signals, while two samples resulted in false-negative detections. Importantly, all five extracts with low-quality or false-negative signals yielded high-quality amplification and, thus, unequivocal positive detections after treatment with the DNeasy PowerClean Pro Cleanup Kit to remove inhibitors. This corresponded to a diagnostic sensitivity of DSE = N_TP_/*N*^+^ = 10/10 = 1 (Table 6). One-to-ten dilutions of the extracts also restored the detection signal, although fluorescence appeared at later cycles compared to extracts treated with the inhibitor removal kit (Fig. 10).

**TABLE 6:** Validation of diagnostic sensitivity using fig samples naturally infected with *Ceratocystis ficicola* (CF)^*a*^

Natural samples (Sicily)	2 µL	1 µL	2 µL of 1:10 diluted	2 µL + inhibitor removal kit
1	21.8 (0.20)	22.6 (0.15)	25.1 (0.14)	22.3 (0.06)
2	**N.D**.	21.8 (0.26)	22.8 (0.14)	20.1 (0.15)
3	22.9 (0.34)	23.5 (0.21)	26.8 (0.53)	23.2 (0.10)
4	22.5 (0.17)	23.4 (0.18)	26.1 (0.29)	23.2 (0.32)
5	29.8 (0.20)	30.6 (0.18)	33.4 (0.25)	30.1 (0.13)
6	24.5 (0.25)	25.9 (0.14)	28.2 (0.22)	25.1 (0.34)
7	27.0 (0.10)	28.1 (0.21)	30.5 (0.37)	27.6 (0.20)
8	23.1 (0.24)	24.0 (0.12)	26.2 (0.19)	23.6 (0.15)
9	**N.D**.	35.2 (0.39)	37.2 (0.36)	34.1 (0.23)
10	22.8 (0.20)	23.9 (0.29)	26.3 (0.10)	23.3 (0.27)

^*a*^ Two-microliter DNA extracts and various dilutions were tested, with or without treatment using the DNeasy PowerClean Pro Cleanup Kit to remove inhibitors. Gray shading indicates collapsed fluorescence signals still interpretable as positive. Detections labeled N.D. (not detected) represent false negatives. Note that diluting template volume and/or using the cleanup kit improved signal quality or restored true positive detection. All samples were confirmed as infected with CF by isolation on culture medium and sequencing.

Natural samples collected from discolored wood of diseased but CF-uninfected trees, and from healthy trees in the Latium region, were used to assess diagnostic specificity. When spiked with CF gDNA, 16 of the 20 extracts produced clear amplification curves and a melt peak identical to that of CF, indicating the absence of significant PCR inhibition. Four extracts required treatment with the inhibitor-removal kit to generate unequivocal amplification curves. Under these established conditions, all extracts tested negative, yielding a best-fit diagnostic specificity, DSP = N_TN_/N⁻ =20/20 = 1 (Table 7).

**TABLE 7:** Natural wood samples from fig trees in the Latium region (Italy) infected with fungi other than *Ceratocystis ficicola* (CF) and used to evaluate diagnostic specificity^*e*^

Wood sample code	Fungal isolate code^*a*^	Fungal microbiota associated^*b*^	Accession no.	Detection of 2 µL wood extract^*c*^
1-H	FIG.16 FIG.17 FIG.18 FIG.19	*Diplodia mutila* *Biscogniauxia mediterranea* *Diplodia* sp. *Diaporthe* sp.	PX992537 (ITS) PX992538 (ITS)	N.D.
2-H	FIG.20 FIG.21 FIG.23	*Fusarium* sp. *Dothiorella viticola* *Neodidymelliopsis* sp.		N.D.
3-H	FIG.27	*Alternaria* sp.		N.D.
4-H	FIG.29	*Alternaria* sp.	PX992540 (ITS)	N.D.
5-H	FIG.10 FIG.11 FIG.12 FIG.14	*Apiospora arundinis* *Alternaria* sp. *Epicoccum* sp. *Pseudopithomyces* sp.		N.D.
6-H	FIG.31 FIG.32	*Sardiniella* sp. *Alternaria* sp.	PX992542 (ITS)	N.D.
7-H	FIG.34	*Diplodia* sp.	PX992544 (ITS)	N.D.
8-H^*d*^		*Diaporthe* sp.		N.D.
9-H^*d*^		*Diaporthe* sp. *Alternaria* sp.		N.D.
10-H		N.A.		N.D.
11-H		N.A.		N.D.
12-H		N.A.		N.D.
13-H		N.A.		N.D.
14-S	FIG.24 FIG.25	*Diaporthe* sp. *Diplodia* sp.	PX992539 (ITS)	N.D.
15-S	FIG.22 FIG.30	*Clonostachys* sp. *Botryosphaeria* sp.	PX992541 (ITS)	N.D.
16-S	FIG.33	*Neofusicoccum* sp.	PX992543 (ITS)	N.D.
17-S	FIG.43	*Diplodia* sp.	PX992546 (ITS)	N.D.
18-S	FIG.35 FIG.36 FIG.44 FIG.45 FIG.46 FIG.47	*Neofusicoccum parvum* *Diaporthe* sp. *Apiospora marii* *Diaporthe cinerascens* *Geosmithia pallida* *Diplodia* sp.	PX992547 (ITS)	N.D.
19-S	FIG.40 FIG.41	*Peniophora* sp. *Neofusicoccum* sp.	PX992545 (ITS)	N.D.
20-S	FIG.51	*Botryosphaeria dothidea*		N.D.

^*a*^ Full fungal isolate code: CREA-DC TPR FIG.X; abbreviated in the table as FIG.X.

^*b*^ N.A., not analyzed for fungal microbiota.

^*c*^ N.D., not detected.

^*d*^ Fungi isolated from samples 8H and 9H were identified morphologically.

^*e*^ Samples coded “H” were collected from healthy trees exhibiting healthy-looking wood, whereas samples coded “S,” symptomatic, were taken from discolored wood of declining trees undergoing desiccation. GenBank accession numbers for sequences used in fungal molecular identification are listed for selected isolates; for the remaining isolates, accession numbers are reported in Table 1.

### Suitability of the amplification protocol for electrophoretic agarose gel analysis

**Fig 11:**
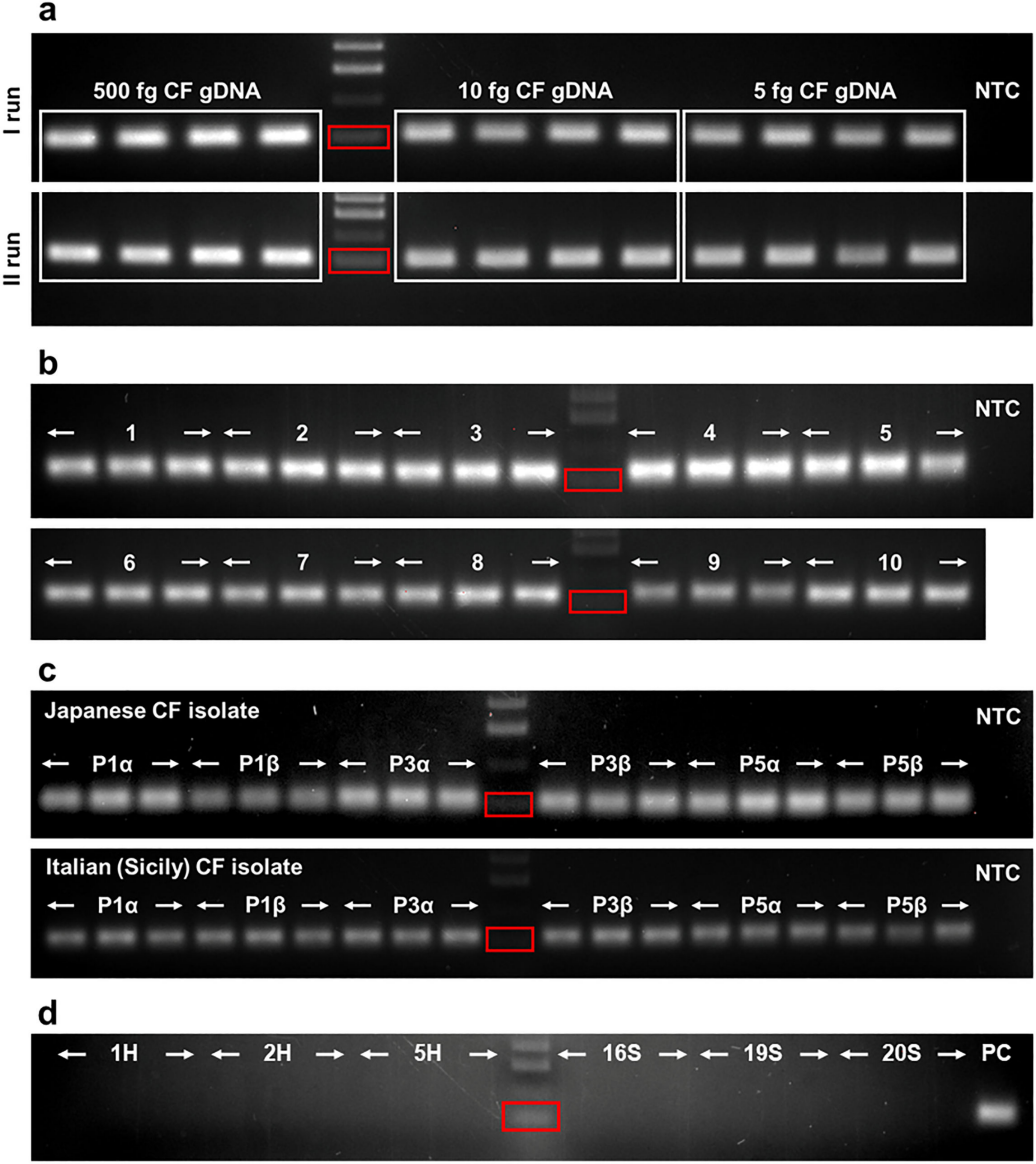
Qualitative validation of the real-time PCR assay by electrophoretic analysis on 2% agarose gel. (**a**) Amplicons from low quantities, including the LoDs, of *Ceratocystis ficicola* (CF) gDNA extracted from axenic culture. The rectangular boundaries have been added to highlight the replicates for each CF gDNA quantity in both real-time PCR runs. (**b**) Amplicons from naturally infected fig samples (see also Table 6). (**c**) Amplicons from fig plants (P) inoculated with Japanese (C1355) and Italian (Sicily) (CPC 44213) CF isolates; for each plant, samples were taken from fully necrotized, heavily colonized areas (α) and from distal regions of the discoloration streak with minimal necrosis and incipient colonization (β) (see also Fig. 6). (**d**) Results of amplifications of wood DNA extracts from fig trees in the Latium region (Italy) infected with fungi other than CF and used to evaluate diagnostic specificity (Table 7); H = healthy, S = symptomatic, PC = positive control. NTC = no template control. Four technical replicates were analyzed for (**a**) and three for (**b**), (**c**), and (**d**). Gels were stained with ethidium bromide (Merck KGaA) for (**a**) and FluoroVue Nucleic Acid Gel Stain (SMOBIO Technology) for (**b**), (**c**), and (**d**). The 100-bp marker is highlighted with a red outline.

Agarose gel electrophoresis revealed clear amplicon bands of the expected size from CF gDNA and DNA extracted from CF-infected samples (Fig. 11). Amplicons generated from CF gDNA at the LoD (10 and 5 fg per PCR) were consistently detected with full repeatability and reproducibility. All samples from naturally infected trees and representative artificially infected trees produced the expected bands, including those obtained from xylem tissues showing minimal or incipient necrosis. In contrast, no amplification products were detected from reactions with DNA extracted from CF-uninfected fig trees, even under overexposed gel conditions.

## DISCUSSION

Diagnostic assessments for (potentially) pathogenic microbes are central to medical and plant pathology investigations, supporting both curative and preventive strategies, as well as in-depth studies of disease biology, epidemiology, and resistance.

In the plant health context, the European and Mediterranean Plant Protection Organization (EPPO) coordinates phytosanitary efforts across the Euro-Mediterranean region to prevent the introduction and spread of harmful pests (https://www.eppo.int/). Following its first reports in EU countries, CF was included in the EPPO Alert List in 2022, reflecting concerns about its potential phytosanitary risk. Although inclusion in the Alert List does not directly mandate regulatory action, it triggers early-warning activities and potential risk evaluation through a Pest Risk Analysis (PRA). Based on the outcomes of the PRA, CF was subsequently added to the EPPO A2 list in 2025 (https://gd.eppo.int/taxon/CERAFC), thereby qualifying as a quarantine pest for which regulation is recommended. As a consequence, National Plant Protection Organizations (NPPOs) are expected to prioritize surveillance and monitoring programs to determine the phytosanitary status of fig plantations throughout the Mediterranean basin. Within this framework, the availability of robust and reliable diagnostic tools is critical, and the present study represents a first step toward addressing this need.

The method described herein is based exclusively on the use of intercalating dyes, with EvaGreen as the primary chemistry and SYBR Green employed for confirmatory testing. The development of a probe-based assay has not been addressed, as the short sequence region between the primers is rich in thymidine stretches and is not conserved among the sequences of the different isolates aligned, due to the presence of SNPs and indels. It may be argued that the use of a probe would have increased the analytical specificity of the method, providing a third annealing zone to distinguish among target and non-target DNA sequences. However, our results demonstrated that the EvaGreen-based assay exhibited a very high degree of specificity, showing no cross-reactivity even in the presence of large amounts of DNA from 50 fungal isolates (representing many fungal taxa) colonizing fig (and olive) wood (Table 1). Remarkably, several of these fungi belong to the Botryosphaeriaceae, Nectriaceae, Bionectriaceae, Diaporthaceae, and Apiosporaceae—families renowned for including species pathogenic to the woody scaffolds of perennial plants, including fig tree. By contrast, unlike probe-based assays, intercalating dye-based assays permit detailed analysis of the melting curve of the amplification product, which can be exploited to discriminate specific amplification products from non-specific ones, as the melting peak temperature uniquely reflects the size and base composition of each amplicon. Therefore, considering that our method combines high specificity and analytical sensitivity with the ability to analyze the melting curve, the development of a probe-based assay was rendered unnecessary. Moreover, the intercalating-based assay is more cost-effective than a probe-based one.

All performance parameters investigated in this work successfully met the criteria for repeatability and reproducibility.

PCR efficiency was evaluated with respect to the wood matrix, the target tissue for the method application. First, with experiments aimed at evaluating the quality of the fluorescent signals, and second with amplification of CF gDNA serial dilutions spiked or not with wood extracts. The results of these experiments clearly indicate that PCR efficiency can be compromised in a dose-dependent manner by compounds present in extracts from necrosis-affected wood. These inhibitory substances are likely derived either from degraded host tissues or from the metabolic activity of microorganisms colonizing necrotic areas. Their concentration appears to be a key determinant of amplification performance, highlighting the quantitative nature of the inhibitory effect.

In contrast, extracts obtained from healthy-looking wood did not interfere with PCR amplification, underscoring the specific impact of necrosis-associated metabolites on reaction efficiency rather than a general matrix effect of woody tissues. Importantly, the implementation of an effective inhibitor removal step ensured optimal amplification efficiency, demonstrating that pre-analytical sample purification represents a critical factor for reliable molecular detection (Fig. 2 and 3; Table 3).

Analytical sensitivity was also evaluated in relation to selectivity, first through the standard curve experiments and then, for intra-laboratory validation, with a high number of replicates at two potential LoDs. In the EvaGreen-based assay, although 5 fg CF gDNA per PCR could be consistently detected with optimal repeatability and reproducibility, 10 fg per reaction was chosen as the operational LoD. In fact, this LoD was shown to ensure robust detection in the 35–36 Ct range while minimizing the risk of overlap with false positives arising from low-level contamination that can occur in laboratory practice at later Cts. The SYBR Green-based assay achieved an even lower operational LoD—3 fg per PCR—while maintaining the same Ct range, underscoring its suitability as a reliable alternative to EvaGreen. In a previous study validating a real-time PCR assay for *C. platani* detection, we showed that the SYBR Green-based supermix—the same employed in the present work—allowed earlier detection of the assay’s LoD compared with EvaGreen and TaqMan chemistries (28). Here, this trend was even more pronounced, as SYBR Green enabled a LoD more than threefold lower than that obtained with EvaGreen, further underscoring the superior analytical sensitivity of this dye, at least in the Bio-Rad formulation.

Overall, these findings indicate that both EvaGreen and SYBR Green assays provide high analytical sensitivity and robustness for CF detection. Importantly, by resorting to the inhibitor removal step, the diagnostic matrix does not adversely affect detection efficiency or sensitivity, even in the consistent presence of inhibitor compounds. This underscores the resilience of the assays and their suitability for reliable detection of low levels of CF biomass in wood-derived samples.

As the final step of the intra-laboratory validation, the method demonstrated optimal performance when applied directly to fig samples that were both naturally and artificially infected with CF (diagnostic sensitivity), as well as to healthy-looking CF-uninfected samples or those infected with pathogens other than CF (diagnostic specificity). The results were fully consistent with those obtained for the homologous parameters, namely inclusivity and exclusivity, providing once again strong evidence for the reliability and robustness of the method.

Specifically, with regard to artificially infected fig trees, testing successfully a large number of samples infected with both a Japanese and an Italian-Sicilian CF isolate confirmed the method’s efficacy under diverse biological conditions: (i) in completely necrotized areas, where PCR inhibitors are presumably more abundant and (ii) at the advancing edge of fungal colonization, distant from the inoculation point, where necrotic streaking was scarcely visible and could potentially challenge the analytical sensitivity of the assay. This would be crucial for timely disease management and surveillance. Notably, lower detection Ct values were obtained from completely necrotized areas, suggesting the absence of significant inhibitory effects and a higher amount of fungal biomass in the areas in which the infection stage was more advanced (Fig. 7). Importantly, in artificially infected samples, successful detection was achieved without the need to process extracts through the inhibitor removal kit, suggesting that inhibitory compounds were either absent or present at negligible levels under artificially managed conditions.

The analysis of naturally infected wood samples further confirmed the robustness of the method under even more challenging conditions, where complex fungal colonization—mainly involving Botryosphaeriaceae, *Fusarium* spp., *Neocosmospora* spp., and *Diaporthe* spp.—had become established. Such colonization likely promoted the accumulation of PCR-inhibitory compounds, even more than in trees artificially infected with just one pathogenic species. Under these conditions, reliable detection was achieved in only half of the samples when 2 µL of extract was used, whereas the others produced low-quality or false-negative signals. The application of mitigation strategies—extract dilution and treatment with the inhibitor removal kit—demonstrated that inhibition could be largely alleviated or even completely eliminated. The treatment with the inhibitor removal kit proved most effective, fully restoring diagnostic signals at earlier Cts and ensuring consistent amplification. Interestingly, applying the inhibitor removal kit to extracts that initially produced optimal fluorescent signals (namely, indicating no inhibitory effects) delayed signals by 0.5–0.7 Ct, suggesting that the treatment for inhibitor removal slightly reduces DNA yield (Table 6). Dilution of the extract may represent a practical alternative in heavily colonized samples; however, in cases of limited fungal colonization, this approach can lower target DNA concentrations below the LoD threshold, resulting in late or undetectable signals (Table 6). These findings highlight the importance of addressing matrix-derived inhibitors in molecular diagnostics of woody tissues to ensure reliable detection under variable microbial colonization scenarios.

Overall, considering the diagnostic sensitivity achieved, we recommend the use of 1 or 2 µL of extract as the optimal template volume for routine application of the method described in this study. Should inhibition be suspected, the use of the aforementioned inhibitor-removal kit—or any other kit proven effective for this purpose—would represent the most appropriate corrective measure. Additionally, running parallel reactions of the same samples spiked with CF gDNA can serve as a reliable internal control to verify the presence of inhibitory effects.

A sensitive and reliable diagnostic assay is also essential for advancing studies on host resistance. In Japan, varying levels of susceptibility (or partial resistance) to *C. ficicola* have been identified among *Ficus carica* cultivars (41–44). More importantly, hybridization with the resistant wild species *F. erecta* Thumb. produced a backcross-derived genotype (“Reikodai 1 go”) suitable as a rootstock, combining strong resistance, good propagation, and compatibility with commercial cultivars (45). Given its performance, the quantitative PCR assay developed in this study would support research on the genetic and physiological bases of susceptibility and resistance, facilitating in-depth studies on “Reikodai 1 go” and accelerating the selection of additional resistant fig varieties.

In just over a decade, several fungal species have been isolated from fig and demonstrated to be agents of bark canker and wood discoloration, to the detriment of stems, branches, and twigs. Within the Botryosphaeriaceae, the following species have been reported for their relevant virulence on fig: *N. parvum* and *N. algeriense* in Italy (46, 47); *Lasiodiplodia theobromae* in Turkey (48), China (49), Korea (50), and in Italy (47); *Neoscytalidium dimidiatum* in California (51, 52), Turkey (53), and Egypt (54). In contrast, *B. dothidea* and *N. luteum* were detected in Italy as secondary components of a fungal community dominated by highly virulent CF and *Neocosmospora* spp. (20, 47).

Within the Nectriaceae, *Neocosmospora* species have been reported as emerging and virulent pathogens of fig: *Neoc. caricae* sp. nov. and *Neoc. metavorans* in Iran (55), and *Neoc. bostrycoides* and *Neoc. persae* in Italy (20, 47).

*Diaporthe cinerascens*, within Diaporthaceae, has long been recognized as a pathogen of fig tree scaffold, causing severe and widespread epidemics in California and Iran (56–60).

Recently, *Stilbocrea banihashemiana* sp. nov. (Bionectriaceae) was described as a novel aggressive fungal pathogen of fig in Iran (61).

Importantly, in this study, a broad panel of isolates representing most of the fungal families and species cited above was subjected to exclusivity testing, and no cross-reactivity was detected, even under stringent conditions, namely, using high gDNA inputs.

An important feature of the decline phenomena observed in figs in Italy, including those with fatal outcomes, is the coexistence of a complex fungal microbiota alongside CF. This assemblage typically includes pathogenic species within the Botryosphaeriaceae, Nectriaceae, and Diaporthaceae (20, 47). Interestingly, during culture isolation, several of the above-mentioned fungal taxa outcompete CF due to their faster growth on artificial media; thus, CF is frequently suppressed and may remain undetected on nutrient agar. As a result, the risk of false negatives for CF is significant when investigating fig declines, potentially leading to overestimating the role of other microorganisms. Therefore, the use of a diagnostic tool, such as the real-time PCR method developed in this study, would be critical for accurate etiological investigations.

In general, the most stringent exclusivity and diagnostic specificity of a detection method would involve the method does not cross-reacting with any other species, apart from the target species, thus excluding also all closely related species, even those specialized for infecting different host species. The ITS region is the most used target for real-time PCR diagnosis of plant pathogens, given its taxonomically related variability and the fact that it is present with multiple copies in the genome of the organisms, which allows for unmatched analytical sensitivity. However, even the ITS region does not always allow the highest specificity degree, and this can be the case for species within Ceratocystidaceae. Two real-time PCR methods have been developed by Pilotti et al. (40) and Luchi et al. (62) for the detection of *C. platani*, targeting the ITS region. Cross-reactivity with some isolates/species of the Ceratocystidaceae was verified for both methods in the laboratory and *in silico* (28). However, the importance represented by these cross-reactions would be strongly minimized by the fact that *Platanus* species are only found infected by *C. platani*. Accordingly, in the present study, the specificity of ITS-based primers within the *Ceratocystidaceae* cannot be assumed a priori, though they did not cross-react with *C. platani*, and warrants further validation. However, *Ceratocystis ficicola* is currently the only *Ceratocystis* species reported to infect fig trees in the EU and in Asia, which substantially reduces the practical relevance of potential cross-reactivity with other *Ceratocystis* species, if any.

Although not within the primary scope of this study, results from the pathogenicity trials and challenges in ITS sequencing of some CF isolates revealed unexpected ancillary findings that warrant brief discussion. (i) *Ceratocystis platani* was able to infect fig plants under experimental conditions, inducing bark cankers and extensive wood discoloration (Fig. 9). Although *C. platani* has been shown to exhibit strong host specialization in reciprocal inoculations with other potential hosts (63, 64), the findings of this study nevertheless demonstrate that the boundaries of host specialization are not absolute and can, unpredictably, be overcome. Though artificial inoculations do not necessarily reflect natural infection processes, targeted diagnostic surveys on fig trees growing in proximity to canker stain foci would be informative. (ii) In this study, reliable ITS sequencing for some CF isolates required cloning, as direct amplicon sequencing yielded ambiguous chromatograms indicative of molecularly heterogeneous amplicons and, consequently, intragenomic ITS variation. High-throughput sequencing (HTS) analyses independently corroborated this interpretation, revealing the coexistence of two distinct ITS ASVs within the genomes of the two isolates from Japan and Sicily. While previously reported in a few LAC *Ceratocystis* isolates (65), our results suggest that this variability may be more widespread within the *Ceratocystidaceae* than currently recognized.

### Conclusions

In conclusion, the performance of the assay presented in this work was evaluated through stringent filters of repeatability and reproducibility. It represents a reliable, sensitive, and highly specific tool for detecting CF in wood across diverse sample conditions. Its resilience to inhibitory compounds and consistent performance in both early and advanced stages of infection, even in the presence of complex microbiota, highlights its versatility and robustness. These attributes make it a useful resource for fundamental research as well as applied phytosanitary monitoring. Future studies should evaluate inter-laboratory reproducibility, as well as its applicability for detecting soil contamination and CF vectoring by fig-infesting beetles, thereby further establishing its utility within integrated diagnostic frameworks for this pathogen.

## Data Availability

All sequence data generated in this study have been deposited in NCBI GenBank under accession numbers PX613668-PX613707 and PX992537–PX992547 (ITS), PX995852–PX995885 (TEF1-α, TUB2) for non-target fungi and PZ052633–PZ052638, PZ052643 (ITS), and PZ066674–PZ066680 (TEF1-α) for CF and *C. platani* isolates. The amplicon sequence variants (ASVs) obtained through the PacBio Sequel II platform in circular consensus sequencing (CCS) mode and classified as ITS were deposited in NCBI GenBank under accession numbers PX994924–PX994927.

## SUPPLEMENTAL MATERIAL

Supplemental material aem.00428-26-s0001.pdf Tables S1 and S2, Text S1, and Fig. S1.

ASM does not own the copyrights to Supplemental Material that may be linked to, or accessed through, an article. The authors have granted ASM a non-exclusive, world-wide license to publish the Supplemental Material files. Please contact the corresponding author directly for reuse.
