# Supplementary material for: Development and validation of real-time PCR for in-wood detection of Ceratocystis ficicola, the agent of canker, wood discoloration, and wilt in the common fig tree (Ficus carica L.)
Source: Appl Environ Microbiol. 2026 Jun 26;92(7):e00428-26. doi: 10.1128/aem.00428-26 (PMC13390406; doi:10.1128/aem.00428-26)
Supplement: Supplemental material — Tables S1 and S2, Text S1, and Fig. S1. [file aem.00428-26-s0001.pdf]

Title:

Development and validation of Real-Time PCR for in-wood detection of Ceratocystis ficicola, the agent of canker, wood discoloration and wilt of common fig tree (Ficus carica L.)

Journal name:

Applied and Environmental Microbiology, AEM

Authors:

Valentina Lumia,[a] Lorenzo Sciarroni,[a] Giorgio Gusella,[b] Daniele Del Corpo,[a] Giuliano Manetti,[a] Erica Cesari,[a] Angela Brunetti,[a] Giancarlo Polizzi,[b] and Massimo Pilotti[a#]

Corresponding author:

Massimo Pilotti

E-mail address:

massimo.pilotti@crea.gov.it

Affiliations:

[a]Research Centre for Plant Protection and Certification (CREA-DC), Rome - Council for Agricultural Research and Economics of Italy (CREA), Italy

[b]Department of Agriculture, Food and Environment, University of Catania, Catania, Italy

Table S1. PCR amplification of DNA regions from target and non-target fungi isolated from fig trees in the Latium, Apulia, and Sicily regions (Italy). **Table S1A** lists the DNA regions amplified and the primers used. **Table S1B** reports the thermal cycling conditions for the amplification of each DNA region.

Table S1A. DNA regions amplified and primers used

**DNA region**	**Forward primer**	**Sequence**	**Reverse primer**	**Sequence**	**Citation**
ITS	ITS5	5'-GGAAGTAAAAGTCGTAACAAGG-3'	ITS4	5'-TCCTCCGCTTATTGATATGC-3'	[30]
TUB2	T1-β tubulin[1]	5'-AACATGCGTGAGATTGTAAGT-3'	Bt2b[2]	5'-ACCCTCAGTGTAGTGACCCTTGGC-3'	[31][1] [32][2]
TUB2 *Biscogniauxia* sp.	Bt2a	5'-GGTAACCAAATCGGTGCTGCTTTC-3'	Bt2b	5'-ACCCTCAGTGTAGTGACCCTTGGC-3'	[32]
TEF1-α	EF688F	5'-CGGTCACTTGATCTACAAGTGC-3'	EF1251R	5'-CCTCGAACTCACCAGTACCG-3'	[33]
TEF1-α *Biscogniauxia* sp.	EF1-728F	5'-CATCGAGAAGTTCGAGAAGG-3'	EF-986R	5′-TACTTGAAGGAACCCTTACC-3′	[34]
TEF1-α *Apiospora* spp.	EF1-728F[3]	5'-CATCGAGAAGTTCGAGAAGG-3'	EF2[4]	5'-GGARGTACCAGTSATCATGTT-3'	[34][3] [35][4]
TEF1-α *Ceratocystis* spp.	EFCF1	5'-AGTGCGGTGGTATCGACAAG-3'	EFCF2	5'-TGCTCACGGGTCTGGCCAT-3'	[36]

Table S1B. Thermal cycling (35 cycles) to amplify DNA regions used for fungal identification (Blast and phylogeny)

**Loci DNA**	**Initial denaturation**	**Denaturation for each cycle**	**Annealing**	**Extension**	**Final extension**
ITS	94°C, 3min	94°C, 30s	50°C, 30s	72°C, 1min 15s	72°C, 10min
TEF1-α *Biscognauxia* sp., Apiospora spp.	94°C, 3min	94°C, 30s	57°C, 40s	72°C, 1min	72°C, 7min
TEF1-α *Ceratocystis* spp.	95°C, 5min	94°C, 30s	52°C, 30s	72°C, 30s	72°C, 10min
TUB2	94°C, 3min	94°C, 30s	50°C, 30s	72°C, 1min	72°C, 7min

References

30. White T, Bruns T, Lee S, Taylor J, Innis M, Gelfand D, Sninsky J. 1990. Amplification and direct sequencing of fungal ribosomal RNA genes for phylogenetics. *in PCR protocols: a guide to methods and applications* 315-322 https://doi.org/10.1016/B978-0-12-372180-8.50042–1.
31. Glass NL, Donaldson GC. 1995. Development of primer sets designed for use with the PCR to amplify conserved genes from filamentous ascomycetes. *Appl Environ Microbiol* 61:1323-1330 DOI:10.1128/aem.61.4.1323-1330.1995.
32. O'Donnell K, Cigelnik E. 1997. Two divergent intragenomic rDNA ITS2 types within a monophyletic lineage of the fungus *Fusarium* are nonorthologous. *Mol Phylogenet Evol* 7:103-116 https://www.sciencedirect.com/science/article/pii/S1055790396903760.
33. Alves A, Crous P, Correia A, Phillips A. 2008. Morphological and molecular data reveal cryptic species in *Lasiodiplodia theobromae*. *Fungal Divers* 28:1–13.
34. Carbone I, Kohn LM. 1999. A method for designing primer sets for speciation studies in filamentous ascomycetes. *Mycol* 91:553-556 https://doi.org/10.1080/00275514.1999.12061051.
35. O'Donnell K, Kistler HC, Cigelnik E, Ploetz RC. 1998. Multiple evolutionary origins of the fungus causing Panama disease of banana: Concordant evidence from nuclear and mitochondrial gene genealogies. *Proc Natl Acad Sci* 95:2044-2049 https://www.pnas.org/doi/10.1073/pnas.95.5.2044.
36. van der Vossen, B.T.H.L.; Westenberg, M.; Botermans, M.; Hodgetts, J.; Cottyn, B.; Warbroek, T.; van Vaerenbergh, J.; Gottsberger, R. 2021. PM 7/129 (2) DNA barcoding as an identification tool for a number of regulated pests. *EPPO Bull* 51:100–143. https://doi.org/10.1111/epp.12724

Table S2. Preliminary evaluation of any effect of inhibition of Real-Time PCR amplification by 1 and 3 µl of healthy and necrotic dried wood matrix (HW and NDW respectively) of fig tree extracted with DNeasy Plant Maxi kit (Qiagen) (I) by alone and with DNeasy Plant Maxi kit plus DNeasy PowerClean Pro Cleanup Kit (Qiagen) (II). Detection *Ct*s of 500 fg and 25 fg of *Ceratocystis ficicola* were used for the evaluation/comparison (in parentheses the standard deviation). This experiment was performed to orient the choice of the extraction modality in order to reduce the inhibition by the matrix as much as possible.

Treatments<br>Extraction Kit	3µl HW + 500fg CF	3µl HW + 25fg CF	1µl HW + 500fg CF	1µl HW + 25fg CF	3µl NDW + 500fg CF	3µl NDW + 25fg CF	1µl NDW + 500fgCF	1µl NDW + 25fg CF	Non-spiked control 500fg CF	Non-spiked control 25fg CF
I	29.7 (0.09)	33.9 (0.35)	29.6 (0.14)	33.7 (0.32)	34.7 (2.07)	38.5 * (0.87)	29.7 (0.31)	33.1 (0.97)	29.49 (0.294)	33.28 (0.290)
I+II	29.4 (0.29)	33.8 (0.35)	29.2 (0.21)	33.6 (0.30)	29.8 (0.32)	33.3 (0.17)	29.4 (0.25)	33.3 (0.36)		

* The value is the average of two technical replicates as one reaction (out of three) was not detected

Text S1. PacBio SMRT sequencing of the ITS region of two representative *Ceratocystis ficicola* isolates, C1355 (Japan) and CPC 44213 (= CBS 149669; Italy). The same OTUs (namely amplified sequence variants, ASV) (OTU-A and OTU-B) were recovered from both isolates. Differences between the two OTU's (SNPs and gaps) are shown based on EMBOSS Needle alignment. Primers designed and used in this study are indicated and mapped onto the alignment.

Simplified OTU table from Biomarker Technologies, BMKGENE, Beijing, China (PacBio SMRT sequencing platform in Circular Consensus Sequencing (CCS) mode). The number indicates the number of consensus circular sequences obtained. OUT = ASV (amplicon sequence variant).

#OTU ID	*Ceratocystis ficicola*<br>C1355<br>(Japan, T.C. Harrington)	*Ceratocystis ficicola*<br>CPC 44213(= CBS 149669)<br>(Italy, G. Polizzi)
OTU-A	15	58303
OTU-B	55475	301

Nucleotide sequences of the OTU's

```
>OTU-A
AAGTCGTAACAAGGTCTCCGTTGGTGAACCAGCGGAGGGATCATTACTGAGTTTTTGTAC
TCTATAAACCATGTGTGAACGTAACGTATCTTGTAATTAAGATAGAGATATTGCTGCTTT
GGTAGTTGGGTGGTTCCCTTCTATAAGGTTTTTCTTCCCACTACCAGCAGCATAATTCTT
CTTTCTTACCACTAAAAACTCTTTTTATTTTTTGTTAGAAATTTGATTTCATTGCTGAGT
GGCATAAACTATAAAAGTTAAAACTTTCAACAACGGATCTCTTGGCTCTAGCATCGATGA
AGAACGCAGCGAAATGCGATAAGTAATGTGAATTGCAGAATTCAGTGAATCATCGAATCT
TTGAACGCACATTGCGCCTGGCAGTATTCTGCCAGGCATGCCTGTCCGAGCGTCATTTCA
CCACTCAAGAACTGTTTTTTTTTCTTGGTGTTGGAGGTCCTGTTCTTCACTGAACAGGCC
GCCGAAATGCATCGGCTGTTCTACTTGCCAGCTCCCCTGTGTAGTACAAAATTTTTACAA
TTTTTACACTTTGAAGCTCTTGTTCAACATGCCGCTAAAAAACCCTCTGTATTCAACTTC
TGTTGGAAACTAATTTTTTTACAAGGTTGACCTCGGATCAGGTAGGAATACCCGCTGAAC
TTAA

>OTU-B
AAGTCGTAACAAGGTCTCCGTTGGTGAACCAGCGGAGGGATCATTACTGAGTTTTTGTAC
TCTATAAACCATGTGTGAACGTAAACGTATCTTGTAATTAAGATAAGAGATATGCTGCTT
TGGTAGTTGGGGGAGTTCCCCTTCTATAAGGTTTTCTTTTCCCACTACCAGCAGTATAAT
TCTTCTTTTTTTTACCACTAAAAACTCTTTTTTTATTTTTTGTTAGAAATTTGATTTCAT
TGCTGAGTGGCATAAACTATAAAAGTTAAAACTTTCAACAACGGATCTCTTGGCTCTAGC
ATCGATGAAGAACGCAGCGAAATGCGATAAGTAATGTGAATTGCAGAATTCAGTGAATCA
TCGAATCTTTGAACGCACATTGCGCCTGGCAGTATTCTGCCAGGCATGCCTGTCCGAGCG
TCATTTCACCACTCAAGAACTGTTTTTTTTTCTTGGTGTTGGAGGTCCTGTTCTTCACTG
AACAGGCCGCCGAAATGCATCGGCTGTTCTACTTGCCAGCTCCCCTGTGTAGTACAAAAT
TTTTACAATTTTTACACTTTGAAGCTCTTGTTCAACATGCCGCTAAAAAAAAACCCTCTG
TATTCAACTTCTGTTGGAAACTAATTTTTTTTTTTTTACAAGGTTGACCTCGGATCAGGT
AGGAATACCCGCTGAACTTAA
```

Primer sequences

```
Fwd-ITS: 5'-TTCCCACTACCAGCAGYATAATTCTTC-3'
Rev-ITS1:5'-CCACTCAGCAATGAAATCAAATTTCTAAC-3' (rc GTTAGAAATTTGATTTCATTGCTGAGTGG)
```

Emboss needle - Pairwise Sequence Alignment (PSA)

https://www.ebi.ac.uk/jdispatcher/psa/emboss_needle/summary?jobId=emboss_needle-I20260128-145039-0111-96815785-p1m

```
# Matrix: EDNAFULL
# Gap_penalty: 10.0
# Extend_penalty: 0.5
#
# Length: 682
# Identity:     659/682 (96.6%)
# Similarity:   659/682 (96.6%)
# Gaps:          19/682 ( 2.8%)
# Score: 3174.5

OTU-A              1 AAGTCGTAACAAGGTCTCCGTTGGTGAACCAGCGGAGGGATCATTACTGA     50
                     ||||||||||||||||||||||||||||||||||||||||||||||||||
OTU-B              1 AAGTCGTAACAAGGTCTCCGTTGGTGAACCAGCGGAGGGATCATTACTGA     50

OTU-A             51 GTTTTTGTACTCTATAAACCATGTGTGAACGT-AACGTATCTTGTAATTA     99
                     |||||||||||||||||||||||||||||||| |||||||||||||||||
OTU-B             51 GTTTTTGTACTCTATAAACCATGTGTGAACGTAAACGTATCTTGTAATTA    100

OTU-A            100 AGAT-AGAGATATTGCTGCTTTGGTAGTTGGGTG-GTT-CCCTTCTATAA    146
                     |||| ||||||| |||||||||||||||||||.| ||| |||||||||||
OTU-B            101 AGATAAGAGATA-TGCTGCTTTGGTAGTTGGGGGAGTTCCCCTTCTATAA    149

OTU-A            147 GGTTTT-TCTTCCCACTACCAGCAGCATAATTCTTC--TTTCTTACCACT    193
                     |||||| |.||||||||||||||||.||||||||||  |||.||||||||
OTU-B            150 GGTTTTCTTTTCCCACTACCAGCAGTATAATTCTTCTTTTTTTTACCACT    199

OTU-A            194 AAAAACTC--TTTTTATTTTTTGTTAGAAATTTGATTTCATTGCTGAGTG    241
                     ||||||||  ||||||||||||||||||||||||||||||||||||||||
OTU-B            200 AAAAACTCTTTTTTTATTTTTTGTTAGAAATTTGATTTCATTGCTGAGTG    249

OTU-A            242 GCATAAACTATAAAAGTTAAAACTTTCAACAACGGATCTCTTGGCTCTAG    291
                     ||||||||||||||||||||||||||||||||||||||||||||||||||
OTU-B            250 GCATAAACTATAAAAGTTAAAACTTTCAACAACGGATCTCTTGGCTCTAG    299

OTU-A            292 CATCGATGAAGAACGCAGCGAAATGCGATAAGTAATGTGAATTGCAGAAT    341
                     ||||||||||||||||||||||||||||||||||||||||||||||||||
OTU-B            300 CATCGATGAAGAACGCAGCGAAATGCGATAAGTAATGTGAATTGCAGAAT    349

OTU-A            342 TCAGTGAATCATCGAATCTTTGAACGCACATTGCGCCTGGCAGTATTCTG    391
                     ||||||||||||||||||||||||||||||||||||||||||||||||||
OTU-B            350 TCAGTGAATCATCGAATCTTTGAACGCACATTGCGCCTGGCAGTATTCTG    399

OTU-A            392 CCAGGCATGCCTGTCCGAGCGTCATTTCACCACTCAAGAACTGTTTTTTT    441
                     ||||||||||||||||||||||||||||||||||||||||||||||||||
OTU-B            400 CCAGGCATGCCTGTCCGAGCGTCATTTCACCACTCAAGAACTGTTTTTTT    449
```

```
OTU-A                442 TTCTTGGTGTTGGAGGTCCTGTTCTTCACTGAACAGGCCGCCGAAATGCA    491
                         ||||||||||||||||||||||||||||||||||||||||||||||||||
OTU-B                450 TTCTTGGTGTTGGAGGTCCTGTTCTTCACTGAACAGGCCGCCGAAATGCA    499

OTU-A                492 TCGGCTGTTCTACTTGCCAGCTCCCCTGTGTAGTACAAAATTTTTACAAT    541
                         ||||||||||||||||||||||||||||||||||||||||||||||||||
OTU-B                500 TCGGCTGTTCTACTTGCCAGCTCCCCTGTGTAGTACAAAATTTTTACAAT    549

OTU-A                542 TTTTACACTTTGAAGCTCTTGTTCAACATGCCGCT---AAAAAACCCTCT    588
                         |||||||||||||||||||||||||||||||||||   ||||||||||||
OTU-B                550 TTTTACACTTTGAAGCTCTTGTTCAACATGCCGCTAAAAAAAAACCCTCT    599

OTU-A                589 GTATTCAACTTCTGTTGGAAACTAA------TTTTTTTACAAGGTTGACC    632
                         |||||||||||||||||||||||||      |||||||||||||||||||
OTU-B                600 GTATTCAACTTCTGTTGGAAACTAATTTTTTTTTTTTTACAAGGTTGACC    649

OTU-A                633 TCGGATCAGGTAGGAATACCCGCTGAACTTAA    664
                         ||||||||||||||||||||||||||||||||
OTU-B                650 TCGGATCAGGTAGGAATACCCGCTGAACTTAA    681
```

Figure S1. Virulence of fungal species inoculated on fig plants used to obtain samples for diagnostic sensitivity and specificity assays. a) Length of necrotic streaks originating at the inoculation point and extending both upward and downward along the stem (measured on longitudinal sections). b) Girdling index, calculated as the ratio between the tangential spread of necrosis and the stem circumference. c) Percentage of necrosis spread in cross sections of vascular tissues at the inoculation point (necrosis deepening was quantified as the proportion of necrosis radial extension in the vascular tissue relative to the total diameter). Red asterisks above bars denote the number of plants that died over the course of the experiment (out of a total of five). CF = *Ceratocysis ficicola*; CP = *Ceratocystis platani*: Neof. vitif. = *Neofusicoccum vitifusiforme*; Neof. parv. = *Neofusicoccum parvum*; Botryo. doth: = *Botryosphaeria dothidea*.

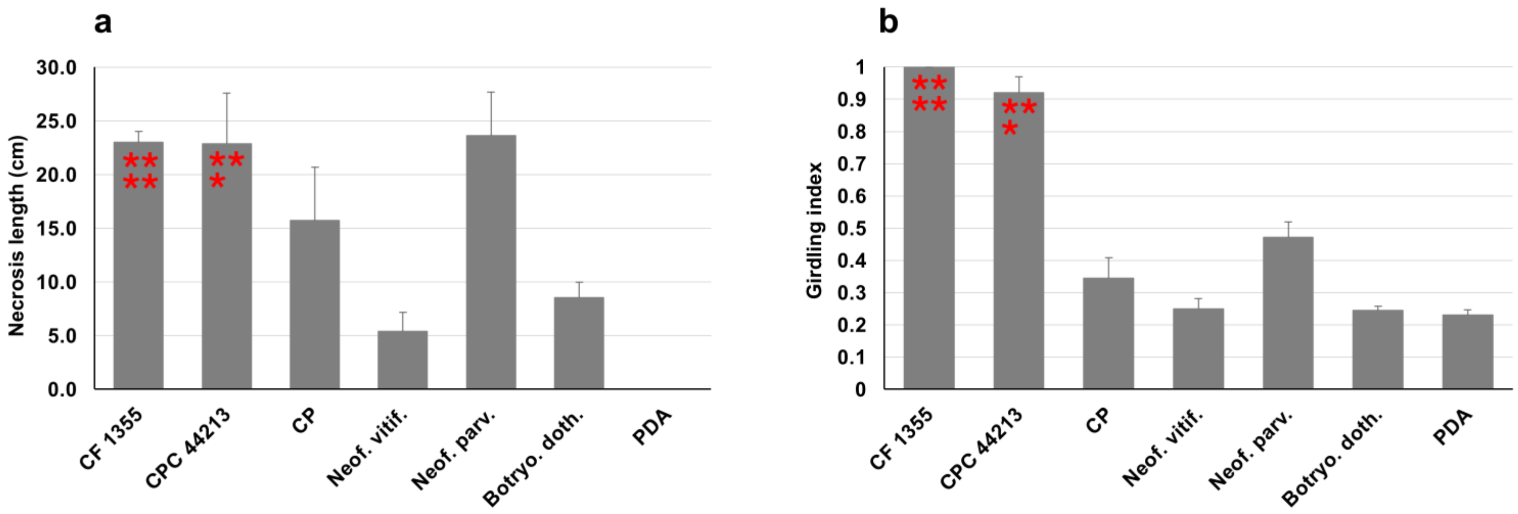

c

CF 1355	CPC 44213	CP	Neof. vitif	Neof. parv.	Botryo. doth.	PDA
100	94	49	0	11	0	0
